# CDK8-Cyclin C Mediates Nutritional Regulation of Developmental Transitions through the Ecdysone Receptor in *Drosophila*

**DOI:** 10.1371/journal.pbio.1002207

**Published:** 2015-07-29

**Authors:** Xiao-Jun Xie, Fu-Ning Hsu, Xinsheng Gao, Wu Xu, Jian-Quan Ni, Yue Xing, Liying Huang, Hao-Ching Hsiao, Haiyan Zheng, Chenguang Wang, Yani Zheng, Alus M. Xiaoli, Fajun Yang, Sarah E. Bondos, Jun-Yuan Ji

**Affiliations:** 1 Department of Molecular and Cellular Medicine, College of Medicine, Texas A&M University Health Science Center, College Station, Texas, United States of America; 2 Department of Chemistry, University of Louisiana at Lafayette, Lafayette, Los Angeles, United States of America; 3 Gene Regulatory Laboratory, School of Medicine, Tsinghua University, Beijing, China; 4 Biological Mass Spectrometry Facility, Robert Wood Johnson Medical School and Rutgers, the State University of New Jersey, Frelinghuysen Road, Piscataway, New Jersey, United States of America; 5 Key Laboratory of Tianjin Radiation and Molecular Nuclear Medicine; Institute of Radiation Medicine, Peking Union Medical College & Chinese Academy of Medical Sciences, Tianjin, China; 6 Department of Medicine, Division of Endocrinology, Diabetes Research and Training Center, Albert Einstein College of Medicine, Bronx, New York, United States of America; 7 Department of Biosciences, Rice University, Houston, Texas, United States of America; Stanford University, UNITED STATES

## Abstract

The steroid hormone ecdysone and its receptor (EcR) play critical roles in orchestrating developmental transitions in arthropods. However, the mechanism by which EcR integrates nutritional and developmental cues to correctly activate transcription remains poorly understood. Here, we show that EcR-dependent transcription, and thus, developmental timing in *Drosophila*, is regulated by CDK8 and its regulatory partner Cyclin C (CycC), and the level of CDK8 is affected by nutrient availability. We observed that *cdk8* and *cycC* mutants resemble EcR mutants and EcR-target genes are systematically down-regulated in both mutants. Indeed, the ability of the EcR-Ultraspiracle (USP) heterodimer to bind to polytene chromosomes and the promoters of EcR target genes is also diminished. Mass spectrometry analysis of proteins that co-immunoprecipitate with EcR and USP identified multiple Mediator subunits, including CDK8 and CycC. Consistently, CDK8-CycC interacts with EcR-USP in vivo; in particular, CDK8 and Med14 can directly interact with the AF1 domain of EcR. These results suggest that CDK8-CycC may serve as transcriptional cofactors for EcR-dependent transcription. During the larval–pupal transition, the levels of CDK8 protein positively correlate with EcR and USP levels, but inversely correlate with the activity of sterol regulatory element binding protein (SREBP), the master regulator of intracellular lipid homeostasis. Likewise, starvation of early third instar larvae precociously increases the levels of CDK8, EcR and USP, yet down-regulates SREBP activity. Conversely, refeeding the starved larvae strongly reduces CDK8 levels but increases SREBP activity. Importantly, these changes correlate with the timing for the larval–pupal transition. Taken together, these results suggest that CDK8-CycC links nutrient intake to developmental transitions (EcR activity) and fat metabolism (SREBP activity) during the larval–pupal transition.

## Abstract 2

During the larval-pupal transition in *Drosophila*, CDK8-CycC helps to link nutrient intake to development by activating ecdysone receptor-dependent transcription and to fat metabolism by inhibiting SREBP-activated gene expression.

## Author Summary

Arthropods are estimated to account for over 80% of animal species on earth. Characterized by their rigid exoskeletons, juvenile arthropods must periodically shed their thick outer cuticles by molting in order to grow. The steroid hormone ecdysone plays an essential role in regulating the timing of developmental transitions, but exactly how ecdysone and its receptor EcR activates transcription correctly after integrating nutritional and developmental cues remains unknown. Our developmental genetic analyses of two *Drosophila* mutants, *cdk8* and *cycC*, show that they are lethal during the prepupal stage, with aberrant accumulation of fat and a severely delayed larval–pupal transition. As we have reported previously, CDK8-CycC inhibits fat accumulation by directly inactivating SREBP, a master transcription factor that controls the expression of lipogenic genes, which explains the abnormal fat accumulation in the *cdk8* and *cycC* mutants. We find that CDK8 and CycC are required for EcR to bind to its target genes, serving as transcriptional cofactors for EcR-dependent gene expression. The expression of EcR target genes is compromised in *cdk8* and *cycC* mutants and underpins the retarded pupariation phenotype. Starvation of feeding larvae precociously up-regulates CDK8 and EcR, prematurely down-regulates SREBP activity, and leads to early pupariation, whereas re-feeding starved larvae has opposite effects. Taken together, these results suggest that CDK8 and CycC play important roles in coordinating nutrition intake with fat metabolism by directly inhibiting SREBP-dependent gene expression and regulating developmental timing by activating EcR-dependent transcription in *Drosophila*.

## Introduction

In animals, the amount of juvenile growth is controlled by the coordinated timing of maturation and growth rate, which are strongly influenced by the environmental factors such as nutrient availability [1,2]. This is particularly evident in arthropods, such as insects, arachnids and crustaceans, which account for over 80% of all described animal species on earth. Characterized by their jointed limbs and exoskeletons, juvenile arthropods have to replace their rigid cuticles periodically by molting. In insects, the larval–larval and larval–pupal transitions are controlled by the interplay between juvenile hormone (JH) and steroid hormone ecdysone [3–7]. *Drosophila* has been a powerful system for deciphering the conserved mechanisms that regulate hormone signaling, sugar and lipid homeostasis, and the molecular mechanisms underlying the nutritional regulation of development [1,2,8–11]. In *Drosophila*, all growth occurs during the larval stage when larvae constantly feed, and as a result their body mass increases approximately 200-fold within 4 d, largely due to de novo lipogenesis [12]. At the end of the third instar, pulses of ecdysone, combined with a low level of JH, trigger the larval–pupal transition and metamorphosis [3,6,13]. During this transition, feeding is inhibited, and after pupariation, feeding is impossible, thus the larval–pupal transition marks when energy metabolism is switched from energy storage by lipogenesis in larvae to energy utilization by lipolysis in pupae.

The molecular mechanisms of ecdysone-regulated metamorphosis and developmental timing have been studied extensively in *Drosophila* [3,5,14,15]. Ecdysone binds to the Ecdysone Receptor (EcR), which heterodimerizes with Ultraspiracle (USP), an ortholog of the vertebrate Retinoid X Receptor (RXR) [16–21]. By activating the expression of genes whose products are required for metamorphosis, ecdysone and EcR-USP are essential for the reorganization of flies’ body plans before emerging from pupal cases as adults. Despite the tremendous progress in our understanding of the physiological and developmental effects of EcR-USP signaling, the molecular mechanism of how the EcR-USP transcription factor interacts with the general transcription machinery of RNA polymerase II (Pol II) and stimulates its target gene expression remains mysterious. EcR is colocalized with Pol II in *Bradysia hygida* and *Chironomus tentans* [22,23]. Although a number of proteins, such as Alien, Bonus, Diabetes and Obesity Regulated (dDOR), dDEK, Hsc70, Hsp90, Rigor mortis (Rig), Smrter (Smr), Taiman, and Trithorax-related (TRR), have been identified as regulators or cofactors of EcR-mediated gene expression [13,24–32], it is unknown how these proteins communicate with the general transcription machinery and whether additional cofactors are involved in EcR-mediated gene expression. In addition, it remains poorly understood how EcR activates transcription correctly after integrating nutritional and developmental cues.

The multisubunit Mediator complex serves as a molecular bridge between transcriptional factors and the core transcriptional machinery, and is thought to regulate most (if not all) of Pol II-dependent transcription [33–40]. Biochemical analyses have identified two major forms of the Mediator complexes: the large and the small Mediator complexes. In addition to a separable “CDK8 submodule”, the large Mediator complex contains all but one (MED26) of the subunits of the small Mediator complex [36,38,41]. The CDK8 submodule is composed of MED12, MED13, CDK8, and CycC. CDK8 is the only enzymatic subunit of the Mediator complex, and CDK8 can both activate and repress transcription depending on the transcription factors with which it interacts [37,42]. Amplification and mutation of genes encoding CDK8, CycC, and other subunits of Mediator complex have been identified in a variety of human cancers [43,44], however, the function and regulation of CDK8-CycC in non-disease conditions remain poorly understood. CDK8 and CycC are highly conserved in eukaryotes [45], thus analysis of the functional regulation of CDK8-CycC in *Drosophila* is a viable approach to understand their activities.

Previously, we have shown that CDK8-CycC negatively regulates the stability of sterol regulatory element-binding proteins (SREBPs) by directly phosphorylating a conserved threonine residue [46]. We now report that CDK8-CycC also regulates developmental timing in *Drosophila* by linking nutrient intake with EcR-activated gene expression. We show that homozygous *cdk8* or *cycC* mutants resemble *EcR* mutants in both pupal morphology and retarded developmental transitions. Despite the elevation of both EcR and USP proteins in *cdk8* or *cycC* mutants, genome-wide gene expression profiling analyses reveal systematic down-regulation of EcR-target genes, suggesting the CDK8-CycC defect lies between the receptor complex and transcriptional activation. CDK8-CycC is required for EcR-USP transcription factor binding to EcR target genes. Mass spectrometry analysis for proteins that co-immunoprecipitate with EcR and USP has identified multiple Mediator subunits, including CDK8 and CycC, and our yeast two-hybrid assays have revealed that CDK8 and Med14 can directly interact with the EcR-AF1 domain. Furthermore, the dynamic changes of CDK8, EcR, USP, and SREBP correlated with the fundamental roles of SREBP in regulating lipogenesis and EcR-USP in regulating metamorphosis during the larval–pupal transition. Importantly, we show that starving the early third instar larvae causes precocious increase of CDK8, EcR and USP proteins, as well as premature inactivation of SREBP; whereas refeeding of the starved larvae reduces CDK8, EcR, and USP proteins, but potently stimulates SREBP activity. These results suggest a dual role of CDK8-CycC, linking nutrient intake to de novo lipogenesis (by inhibiting SREBP) and developmental signaling (by regulating EcR-dependent transcription) during the larval–pupal transition.

## Results

### The *cdk8* and *cycC* Mutants Are Defective in the Larval–Pupal Transition

**Fig 1:**
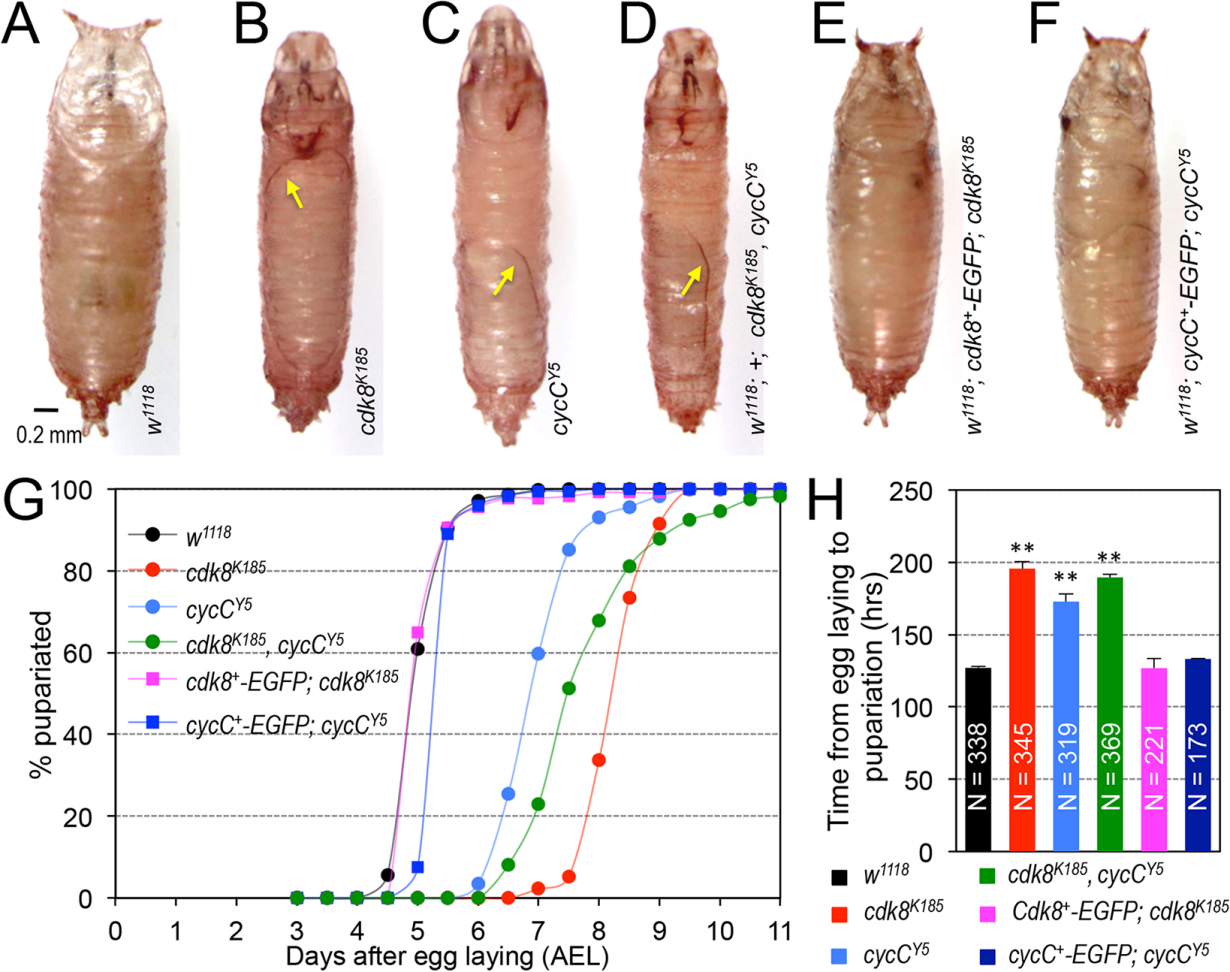
Loss of CDK8 or CycC leads to defective pupal morphology and delayed larval–pupal transition. (A–D) Compared to the control (A; *w* ^*1118*^), *cdk8* (B; *w* ^*1118*^ *; +; cdk8* ^*K185*^) and *cycC* (C; *w* ^*1118*^ *; +; cycC* ^*Y5*^) single mutants, as well as the *cdk8-cycC* double mutants (D; *w* ^*1118*^ *; +; cdk8* ^*K185*^, *cycC* ^*Y5*^) fail to evert their anterior spiracles and the prepupae are partially separated from the pupal case (arrows). Scale bar in (A): 0.2mm. (E and F) These defects were rescued in transgenic lines carrying genomic fragments of wild-type *cdk8* ^*+*^ (E; *w* ^*1118*^ *; cdk8* ^*+*^ *-EGFP; cdk8* ^*K185*^) or *cycC* ^*+*^ (F; *w* ^*1118*^ *; cycC* ^*+*^ *-EGFP; cycC* ^*Y5*^), respectively. (G) The larval-to-pupal transition was analyzed by observing the percentage of pupariated animals after egg laying (AEL) once every 12 hr. (H) The time from egg deposition to pupariation in *cdk8*, *cycC* mutants, and the rescued animals. * *p* < 0.05; ** *p* < 0.01 based on *t*-tests. Underlying numerical data and statistical analysis for Fig 1G and 1H can be found in S1 Data.

The *Drosophila cdk8* and *cycC* genes were originally identified based on the function and sequence conservation to their yeast and human orthologs [47–49]. *cdk8* ^*K185*^ and *cycC* ^*Y5*^ are null alleles that delete part of *cdk8* (882 bp) and all of *cycC* (2,733 bp), respectively, and the homozygous mutants are both prepupal lethal [50]. Mutant animals are able to develop to prepupae, likely due to maternally loaded CDK8 and CycC mRNAs and proteins, because embryos derived from the *cycC* ^*Y5*^ germline clones are smaller and are embryonic lethal without proper denticle formation (S1 Fig). In contrast to the wild-type pupae (Fig 1A), 96% of *cdk8* ^*K185*^ (Fig 1B) and 97% of *cycC* ^*Y5*^ (Fig 1C) homozygous mutants fail to evert their anterior spiracles (quantified in S2A Fig), and prepupae of both mutants are partially separated from their pupal cases (arrows in Fig 1B and 1C). In addition, pupariation is delayed by about 2 to 3 d in the *cdk8* and *cycC* mutants (Fig 1G and 1H).

To investigate the effects of CDK8-CycC on developmental timing, we first analyzed the *cdk8-cycC* double mutant animals by genetically combining the *cdk8* ^*K185*^ and *cycC* ^*Y5*^ null alleles in the same organism. The phenotypes in the *cdk8-cycC* double mutant animals were similar to *cdk8* or *cycC* single mutants, including pupal morphology (Figs 1D and S2A), delayed pupariation (Fig 1G and 1H), and prepupal lethality. The levels of *cdk8* and *cycC* mRNA (S2B Fig) and their protein products (S2C Fig) are diminished in *cdk8* or *cycC* single and double mutant larvae when assayed at the third instar larval stage (L3). The protein level of CycC is significantly reduced in *cdk8* and *cycC* mutants, but the level of CDK8 is not affected in *cycC* mutants (S2C Fig), thus the stability of CycC is dependent on CDK8 but not vice versa.

To validate that the loss of CDK8-CycC causes the defects in pupal morphology and development, we tested whether the mutant phenotypes could be rescued by expression of wild-type CDK8 or CycC. Since CDK8 and CycC form the CDK8 submodule with MED12 and MED13 in a 1:1:1:1 stoichiometry [51], proper dosage of these four subunits is critical for the formation and function of a viable CDK8 sub-module. To ensure proper expression levels and patterns, we generated transgenic flies using genomic fragments of *cdk8* and *cycC* loci with EGFP tags at their C-termini (S3 Fig). The X-ray crystal structure of human CDK8-CycC complex demonstrates that the C-termini of CDK8 and CycC are not involved in their interaction [52], thus epitope tags fused to C-termini were expected to avoid functional disruption of the CDK8-CycC complex. These constructs were transposed to chromosome 2; the transgenic flies are referred to as “*cdk8* ^*+*^ *-EGFP*” or “*cycC* ^*+*^ *-EGFP*” for simplicity. We genetically combined these transgenes with *cdk8* or *cycC* null alleles, thus CDK8 or CycC proteins were tagged with EGFP in the rescued animals (“*w* ^*1118*^ *; cdk8* ^*+*^ *-EGFP; cdk8* ^*K185*^” for *cdk8*-rescued animals, and “*w* ^*1118*^ *; cycC* ^*+*^ *-EGFP; cycC* ^*Y5*^” for *cycC*-rescued animals). The genotypes of the rescued adult animals were validated by PCR analysis (S3 Fig). Importantly, these transgenic lines rescue both the pupal morphology (Figs 1E and 1F, and S2A) and developmental timing (Fig 1G and 1H). The rescued animals are no longer prepupal lethal, and they emerge as adult flies. These observations indicate that CDK8 and CycC are required for proper developmental transitions in *Drosophila*.

### The *cdk8* and *cycC* Mutants Are Defective in EcR-Dependent Gene Expression

**Fig 2:**
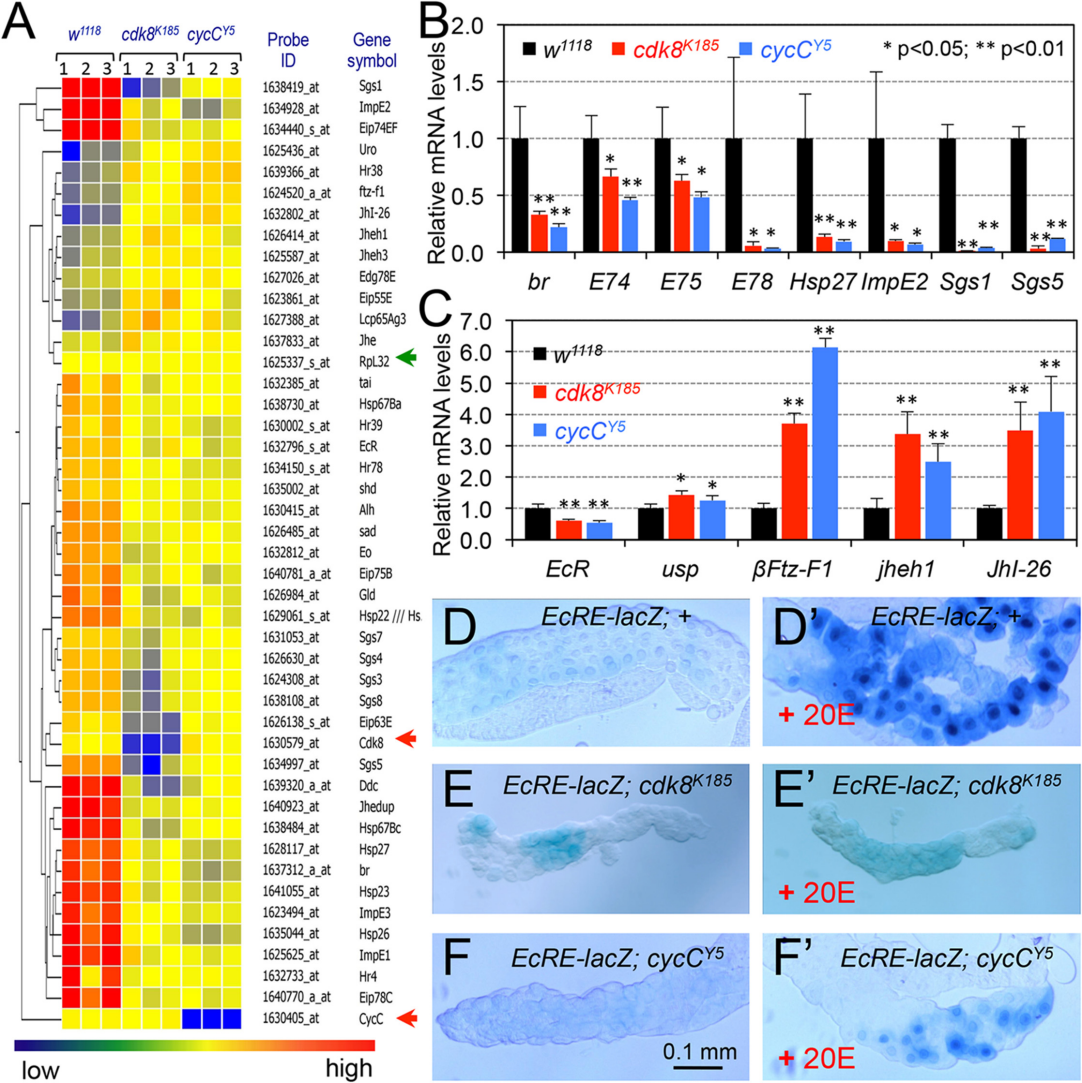
Expression of EcR-target genes is defective in *cdk8* or *cycC* mutant larvae. (A) Microarray analyses of genes whose products are related to functions of the ecdysone and JH. The *RpL32* (*Rp49*, green arrow head) serves as the negative control, and *cdk8* and *cycC* are positive controls (red arrow heads). (B and C) The microarray data were validated by qRT-PCR in the L3 wandering larvae. * *p* < 0.05; ** *p* < 0.01 based on *t*-tests. (D–F) Compared to the control (salivary glands in D and D’ are from the same larvae; genotype: *w* ^*1118*^ *; EcRE-lacZ;+*), the expression of *EcRE-lacZ* was significantly reduced in *cdk8* (E versus E’, from the same larvae; genotype: *w* ^*1118*^ *; EcRE-lacZ; cdk8* ^*K185*^) and *cycC* (F versus F’, from the same larvae; genotype: *w* ^*1118*^ *; EcRE-lacZ; cycC* ^*Y5*^) mutant genetic backgrounds. Scale bar in (F): 0.1mm. Underlying numerical data and statistical analysis for Fig 2B and 2C can be found in S1 Data.

The phenotypes of *cdk8* and *cycC* mutants (Fig 1) are reminiscent of loss-of-function alleles of *EcR-B1*, the major EcR isoform that controls the larval-to-pupal transition [53]. The *EcR* gene encodes three isoforms (EcR-A,-B1, and-B2) that are expressed in tissue- and developmental stage-specific manners [21,53,54]. To test the possibility that CDK8-CycC and EcR-B1 regulate similar molecular events that control the developmental transitions, we first examined whether the expression of EcR target genes was affected in *cdk8* or *cycC* mutants. By mining the microarray data that we published previously [46], we analyzed the mRNA levels of 67 genes whose products are related to the ecdysone and JH activities as reported in the literature (S1 Table) [3,6]. In *cdk8* or *cycC* mutants, the mRNA levels of 33 of these genes are significantly decreased whereas mRNA levels of 10 of these genes are increased more than 1.5-fold compared to the control. Most of the down-regulated genes are EcR-activated genes, while most of the up-regulated genes respond to JH activity (Fig 2A and S1 Table).

We used qRT-PCR assays to verify the levels of several well-characterized direct target genes of EcR, such as *broad*, *E74*, *E75*, *E78*, *Hsp27* (*Heat shock protein 27*), *ImpE2* (*Ecdysone-inducible gene E2*), *Sgs1* (*Salivary gland secretion 1*), and *Sgs5* [3,6]. As shown in Fig 2B, the expression of these EcR-activated genes was significantly reduced in L3 wandering *cdk8* and *cycC* mutants. There was a small reduction of *EcR* mRNA levels, but a mild increase of *usp* mRNA levels, in the *cdk8* and *cycC* mutants (Fig 2C). EcR normally represses the expression of the mid-prepupal gene *βFtz-F1* during the larval stage [15,55]; however, the expression of *βFtz-F1* was dramatically increased in the *cdk8* and *cycC* mutant larvae (Fig 2C), suggesting that the function of EcR is disrupted in the *cdk8* and *cycC* mutants. Likewise, the levels of *jheh1* (*JH-epoxide hydrolase*) and *JhI-26* (*JH-inducible protein 26*) were significantly increased in *cdk8* and *cycC* mutants (Fig 2C). JHEH1 is involved in the catabolic processing of JH, while the expression of *JhI-26* is induced by either methoprene or JH III [56]. Therefore, the expression of both EcR and JH-regulated genes was deregulated in *cdk8* and *cycC* mutants, consistent with the developmental retardation phenotype (Fig 1G and 1H).

To test whether ecdysone-induced EcR target gene expression was generally compromised in *cdk8* or *cycC* mutants, we analyzed the effect of *cdk8* or *cycC* mutation on the expression of the multimerized *hsp27 EcRE* (*ecdysone response element*)-*lacZ* reporter [21,54]. In response to the treatment of 20-hydroxyecdysone (20E), the most biologically potent EcR ligand, β-galactosidase activity was induced in the control salivary gland cells as expected (Fig 2D versus 2D’). However, this response was significantly compromised in salivary glands from the *cdk8* (Fig 2E’) and *cycC* (Fig 2F’) homozygous mutants; the glands from the same animals (Fig 2E and 2F, respectively) were used as the controls. These results were consistent with reduced expression of EcR target genes in *cdk8* or *cycC* mutants (Fig 2A and 2B).

### Effects of *cdk8* or *cycC* Mutation on Biosynthesis of Ecdysteroids

**Fig 3:**
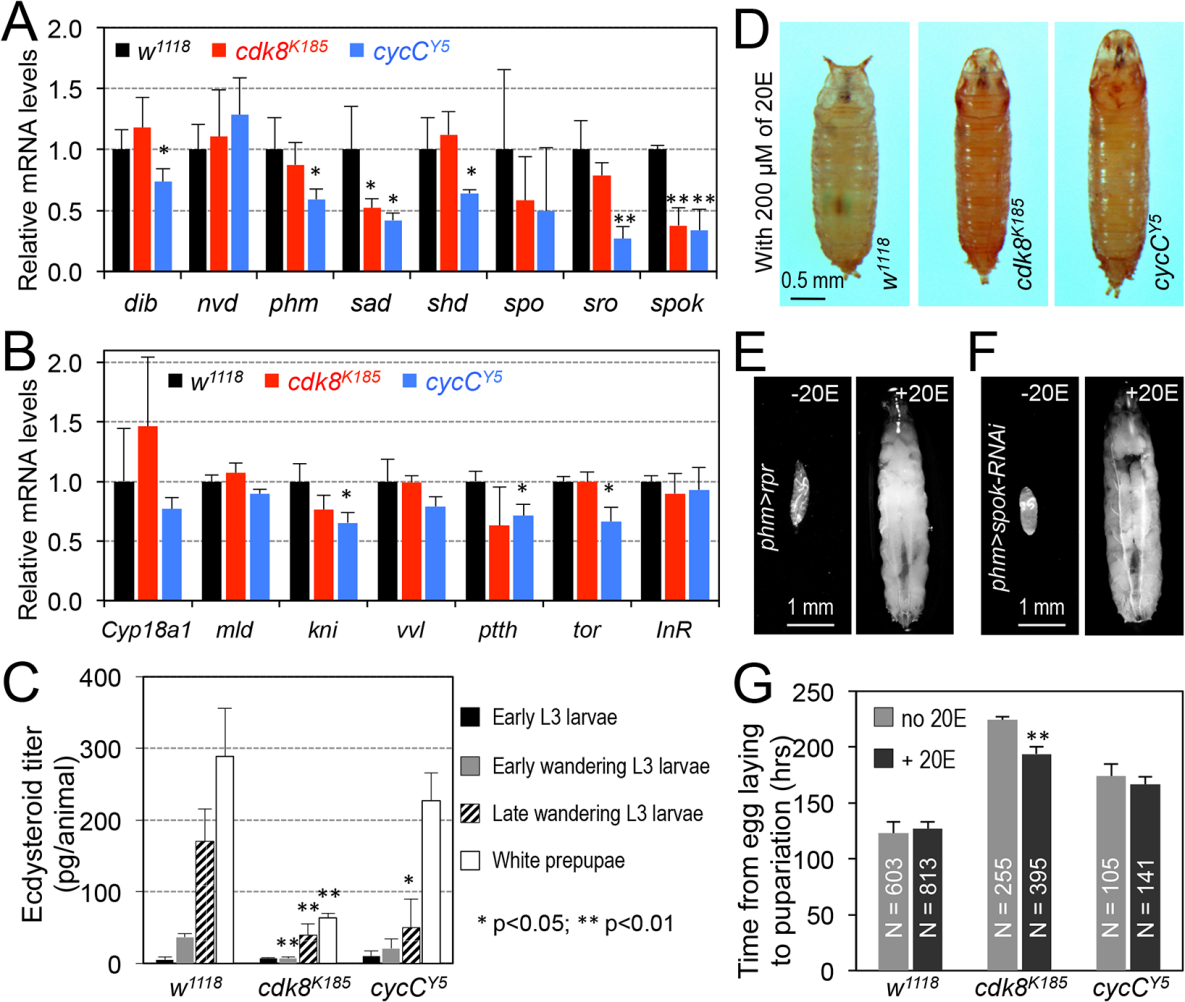
The effects of *cdk8* or *cycC* mutation on biosynthesis of ecdysteroids. (A) Expression of the Halloween genes analyzed by qRT-PCR in the L3 wandering larvae of the following genotypes: *w* ^*1118*^ (black bars), *cdk8* ^*K185*^ (red), and *cycC* ^*Y5*^ (blue). (B) Expression of genes encoding factors that are involved in regulating degradation of 20E (*Cyp18a1*), expression of the Halloween genes (*mld*, *kni*, and *vvl*), the neuropeptide prothoracicotropic hormone PTTH (*ptth*), and metabolism (*tor* and *InR*) in *cdk8* and *cycC* mutant L3 wandering larvae. (C) Ecdysteroid titers determined by ELISA in *cdk8* and *cycC* mutant animals from early L3 larvae to white prepupal stage. (D) Supplying the EcR ligand 20-hydroxyecdysone (20E, 200 μM) in food did not rescue the aberrant morphology of the *cdk8* and *cycC* mutants. (E and F) Supplying 20E in food efficiently rescued the developmental arrest caused by either ablating PG cells using PG-specific (*phm-Gal4*) expression of *reaper* (E) or PG-specific knockdown of *spok* using RNAi (F). (G) Quantification of the effect of 20E (200 μM) on the time from egg deposition to pupariation in *cdk8* and *cycC* mutants. * *p* < 0.05; ** *p* < 0.01 based on *t*-tests. Underlying numerical data and statistical analysis for Fig 3A, 3B, 3C, and 3G can be found in S1 Data.

Both the ecdysone ligand and the EcR-USP transcription factor complex are required for the expression of EcR target genes. 20E directly binds to the ligand-binding domain (LBD) of EcR, which then activates EcR target gene expression [3,6,13]. Therefore, down-regulated expression of EcR target genes in *cdk8* and *cycC* mutants may be due to defective biosynthesis of 20E, or defects in EcR-activated transcription. To test whether the biosynthesis of 20E is defective in *cdk8* and *cycC* mutants, we analyzed the expression of enzymes that are required for the biosynthesis of 20E, such as *nvd* (*neverland*, encoding an oxygenase-like protein) and a family of cytochrome P450 enzymes including *dib* (*disembodied*), *phm* (*phantom*), *sad* (*shadow*), *shd* (*shade*), *spo* (*spook*), and *spok* (*spookier*), collectively known as the Halloween genes [57–59]. The expressions of *sad* and *spok* were decreased in the *cdk8* and *cycC* mutants, but the expression of *nvd* and other Halloween genes were not significantly affected (Fig 3A). In addition, we analyzed the mRNA levels of *cyp18a*, which encodes a cytochrome P450 enzyme involved in degradation of 20E [60], and a few genes encoding factors involved in regulating the expression of the Halloween genes, such as *mld*, *kni*, and *vvl* [61]. As shown in Fig 3B, no obvious changes of these genes were observed in both *cdk8* and *cycC* mutants. Nevertheless, reduction of *sad* and *spok* in *cdk8* and *cycC* mutants indicates that the biosynthesis of ecdysone may be defective in the *cdk8* and *cycC* mutants.

Next, we measured the levels of ecdysteroids in *cdk8* and *cycC* mutants from early L3 larval stage to white prepupal (WPP) stage. Compared to the control, the levels of ecdysteroids are significantly lower in *cdk8* mutant animals during the wandering L3 and WPP stages than the control, while the levels of ecdysteroids are lower in *cycC* mutants only during the late wandering stage (Fig 3C). Nevertheless, the levels of ecdysteroids are continuously increased from early L3 to WPP stage in both *cdk8* and *cycC* mutants, indicating that the biosynthesis of ecdysteroids is compromised, but not completely abolished, in these mutants.

To further determine whether the developmental retardation in *cdk8* and *cycC* mutants is caused by impaired 20E biosynthesis, we fed the homozygous mutants with fly food supplemented with 200 μM of 20E, which is an established approach used to examine whether developmental defects are caused by mutations that disrupt biosynthesis of ecdysteroid [62–64]. However, the defective prepupal morphology of the *cdk8* and *cycC* mutants was not rescued (Fig 3D). In contrast, food supplement of 20E rescued animals with prothoracic gland (PG) cells ablated by PG-specific expression of *reaper* gene (Fig 3E), which triggers apoptosis, or animals with *spok* specifically depleted in the PG (Fig 3F) using a PG-specific driver (*phm-Gal4*). Therefore, supplement of 20E to larvae depleted in factors required for ecdysone biosynthesis rescues their developmental delay [61]. Consistent with reduced ecdysteroids level in *cdk8* mutants (Fig 3C), feeding *cdk8* mutant with 200 μM of 20E in food had a mild effect on the time from egg deposition to pupariation compared to the control (Fig 3G). However, feeding *cycC* mutants with 20E had no effect on their developmental delay, also consistent with the weaker effect of *cycC* mutation on ecdysteroids levels (Fig 3C and 3G). Importantly, the larval–pupal transition is still significantly retarded in *cdk8* or *cycC* mutants, even when fed with 20E (Fig 3G). Other concentrations of 20E in food, ranging from 2 μM to 2mM, also failed to rescue the developmental defects of the *cdk8* and *cycC* mutants (S4 Fig). These results suggest that defective biosynthesis of 20E alone is not sufficient to explain the developmental defects of *cdk8* or *cycC* mutants, which is consistent with the strongly compromised effects of 20E on *EcRE-lacZ* expression in salivary gland cells in *cdk8* and *cycC* mutants (Fig 2E and 2E’ and 2F and 2F’). Considering that CDK8 and CycC function as subunits of the transcription cofactor Mediator complex, which is known to regulate the transcriptional activity of several nuclear hormone receptors in mammals [65,66], the most likely scenario is that the *cdk8* and *cycC* mutants are defective in the regulation of EcR-dependent gene expression in peripheral tissues, in addition to impairing ecdysone biosynthesis in the PG.

### EcR and USP Are Accumulated in the Nuclei in *cdk8* or *cycC* Mutants

**Fig 4:**
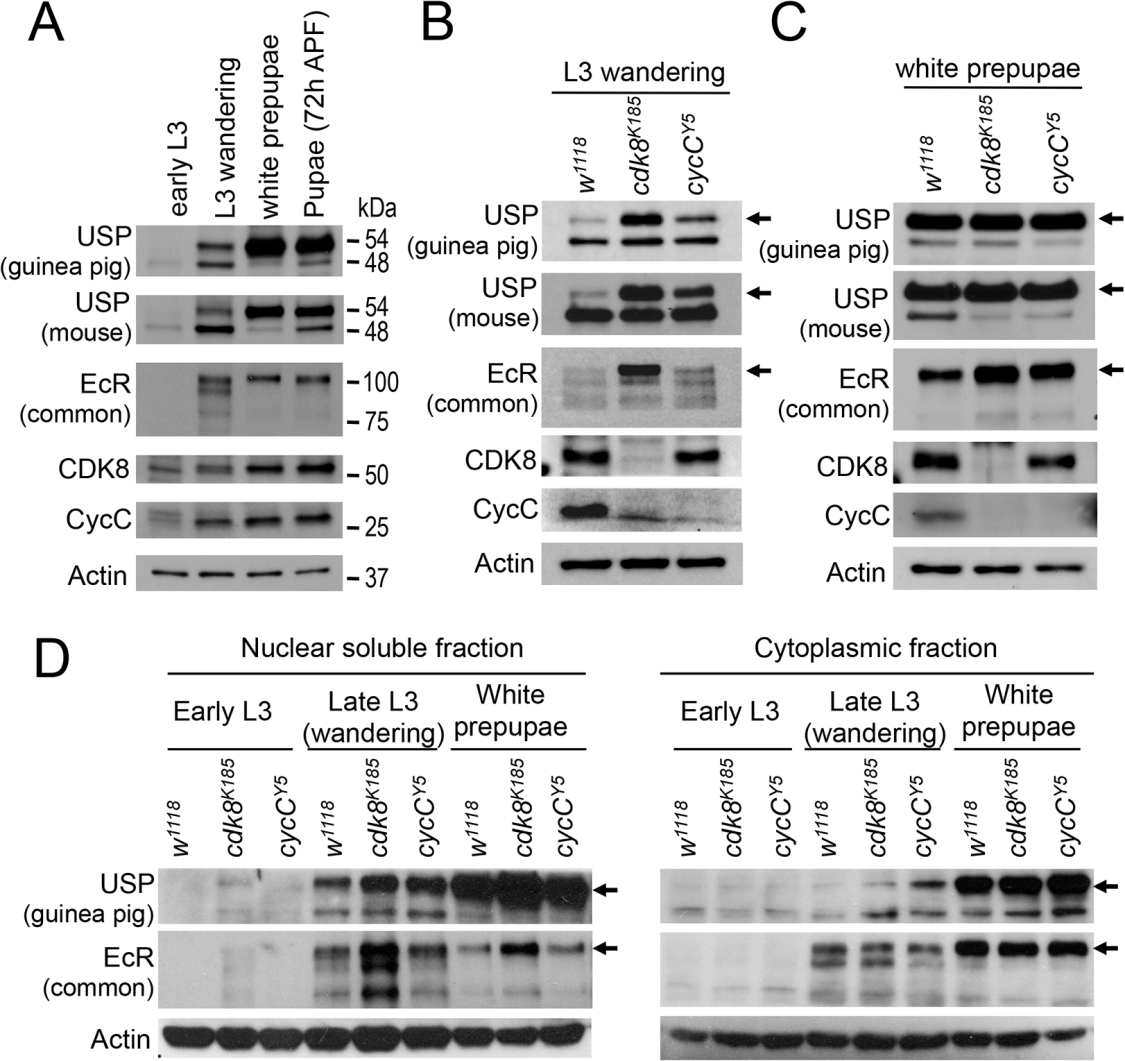
The levels and subcellular distribution of EcR and USP in *cdk8* and *cycC* mutants in the third instar larvae and pupae. (A) Western blot of EcR and USP in wild-type animals in early L3 (84 hr AEL), L3 wandering (112 hr AEL), white prepupal (120 hr AEL), and pupal stages (72 hr APF). (B and C) Western blot analyses of the protein levels of EcR, USP, CDK8, and CycC in *cdk8* and *cycC* mutants at the L3 wandering stage (B) and the white prepupal stage (C). The arrows in USP blots mark the 54 kDa full-length USP, and the arrows in EcR blots indicate the EcR-B1 isoform. (D) Western blot of EcR and USP in the nuclear and cytoplasmic fractions from early third instar larvae (L3) to white prepupal stages in *cdk8* or *cycC* mutants.

To understand how loss of CDK8 or CycC reduced EcR-target gene expression, we tested whether the protein levels of EcR and USP were affected in *cdk8* and *cycC* mutants. Since the major defects occurred during the larval–pupal transition, we first analyzed the expression of EcR, USP, CDK8, and CycC in wild-type larval and pupal extracts from the early L3 larvae (84hr AEL [after egg laying]), the late L3 wandering larvae (112hr AEL), at pupariation (0 hr APF [after puparium formation]), and pupae (72 hr APF). The level of EcR-B1 (105 kDa) was low in the early L3 stage, but was significantly increased from the wandering to 72 hr APF stage (Fig 4A), which lags the temporal expression profile of *EcR-B* mRNA [54,67]. The monoclonal antibody against USP (AB11) recognizes two forms of USP: the 54 kDa full-length USP protein and the 48 kDa truncated USP that lacks the most N-terminal portion [68–70]. The truncated USP is proposed to derive from alternative usage of translation start sites or protease cleavage [68,69,71]. We detected both isoforms of USP, and observed that the levels of both isoforms, particularly the full-length USP, are significantly increased during the pupal stages (Fig 4A). To facilitate biochemical analyses of USP (see below), we generated a polyclonal USP antibody in guinea pig. Similar to the USP monoclonal antibody [72,73], this new polyclonal antibody also specifically recognizes the two isoforms of USP (S5A Fig), and reveals a similar expression pattern of USP during development (Fig 4A). Interestingly, the protein levels of CDK8 and CycC are increased after the L3 wandering stage (Fig 4A).

Because the major changes in EcR, USP, CDK8, and CycC levels occurred during the L3 larval to pupal transition (Fig 4A), we performed our subsequent analyses of *cdk8* and *cycC* mutants at the L3 wandering stage and the white prepupal stage. During the L3 wandering stage, the level of the full-length USP protein was significantly increased in *cdk8* and *cycC* mutants (Fig 4B). The level of EcR-B1 was increased in the mutants, particularly in the *cdk8* mutant larvae (Fig 4B). In white prepupae, the level of EcR-B1 was also significantly increased in *cdk8* and *cycC* mutants, but the level of full-length USP was similar to the control (Fig 4C). Thus, the total protein levels of EcR are higher in *cdk8* and *cycC* mutants than in controls during the L3 wandering stage and the white prepupal stage, while the total protein levels of USP are higher in *cdk8* and *cycC* mutants during the L3 wandering stage.

Since the expression of the EcR-USP target genes is reduced in *cdk8* and *cycC* mutants (Fig 2), we did not expect that protein levels of EcR and USP would be increased in mutants at the same stage (Fig 4B and 4C). Thus we examined whether the subcellular distribution of EcR or USP were affected in *cdk8* or *cycC* mutants by performing immunostaining of the salivary glands from the L3 wandering larvae. Both EcR (S6A Fig) and USP (S6A’ Fig) were localized in the nuclei in wild-type salivary glands. In *cdk8* and *cycC* mutant glands, the levels of EcR (S6B and S6C Fig) and USP (S6B’ and S6C’ Fig) in both nucleus and cytoplasm appear to be slightly elevated compared to the control (S6A and S6A’ Fig, respectively), which is supported by quantification of these images using ImageJ (S6D and S6E Fig). These results suggest that the cytoplasmic levels of EcR or USP and nuclear levels of USP were increased in *cdk8* and *cycC* mutants.

Since immunostaining is not a robust quantitative approach, we fractionated nuclear soluble and cytoplasmic fractions of total proteins and analyzed the levels of EcR and USP by Western blot. The full-length USP protein was significantly increased in the nuclear soluble fraction of samples from *cdk8* and *cycC* mutants during the late L3 wandering and WPP stages (Fig 4D, left panel). In addition, the nuclear EcR levels were higher in *cdk8*, and to a lesser extent, *cycC* mutants, than the control at the late L3 and WPP stages (Fig 4D). In contrast, when analyzing the cytoplasmic fraction from the early L3 to WPP stage, we observed that USP level was a bit higher in *cdk8* and *cycC* mutants than the control, but there was no obvious difference in cytoplasmic EcR levels (Fig 4D, right panel). Nevertheless, these analyses show that the increased EcR and USP proteins in *cdk8* or *cycC* mutants are predominantly localized in the nuclei during early and late L3 stage, suggesting that the subcellular localization of EcR and USP are not affected in the *cdk8* or *cycC* mutants.

Interestingly, the salivary gland cells in the *cdk8* and *cycC* mutants are smaller than the control of the same stage (compare S6B and S6C with S6A Fig), in addition to weaker DAPI staining (S6B” and S6C” Fig, compared to the control in S6A” Fig). The sizes of salivary gland cells positively correlate to the DNA content [74]. The giant polytene chromosomes are produced from successive rounds of DNA endoreduplication. At the molecular level, DNA endoreduplication is controlled by periodical E2F1-activated expression of *cyclin E* (*cycE*) gene followed by transient degradation of E2F1 protein, which is mediated by the CRL4 (CDT2) ubiquitin ligase [75]. Previously, we have reported that CDK8-CycC negatively regulates E2F1 activity in *Drosophila* [76]. As measured by qRT-PCR, the levels of E2F1 targets genes, such as *CG7670*, *cycE*, *MCM5*, *mus209* (encoding PCNA), *Orc5*, *rnrL*, and *stg*, are indeed significantly increased in the *cdk8* or *cycC* mutant salivary glands (S7 Fig). These results suggest that the smaller salivary glands in the *cdk8* and *cycC* mutants are likely caused by dysregulated E2F1 activity and endoreduplication.

### CDK8-CycC Is Required for the Recruitment of EcR-USP to its Target Genes

**Fig 5:**
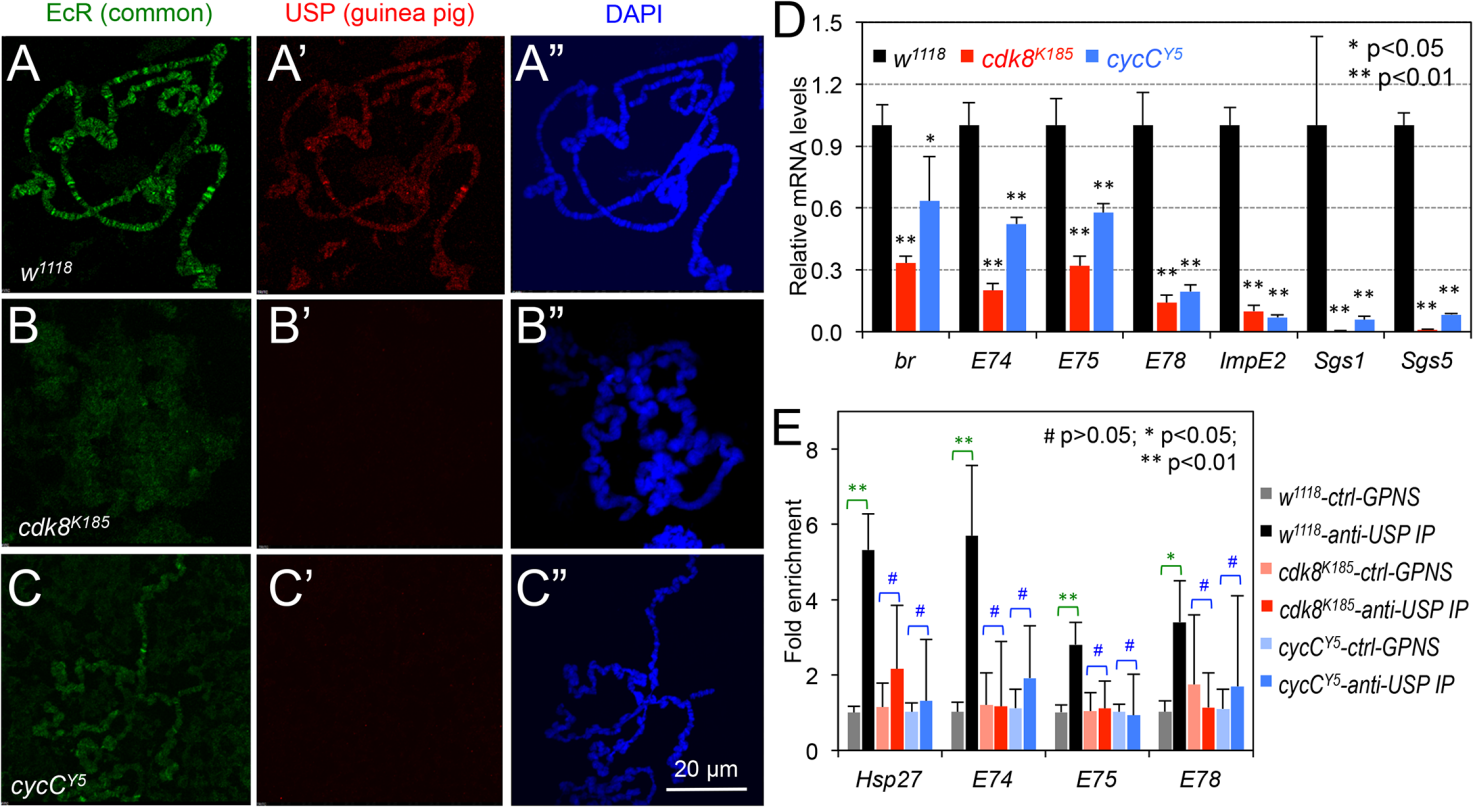
The levels of EcR and USP on polytene chromosome correlate with the expression of EcR target genes in salivary glands. (A–C) Immunostaining of EcR (A, B, and C) and USP (A’, B’, and C’) in *w* ^*1118*^ (control), *cdk8* ^*K185*^, and *cycC* ^*Y5*^ polytene chromosomes, which were also stained with DAPI (A”, B”, and C”). Scale bar in (C”): 20μm. (D) Quantification of EcR target gene expression in salivary glands by qRT-PCR. (E) ChIP assay of USP binding to EcR target gene promoters in the *w* ^*1118*^, *cdk8* ^*K185*^, and *cycC* ^*Y5*^ mutant larvae. Ctrl: control; GPNS: guinea pig normal serum; IP: immunoprecipitation. * *p* < 0.05; ** *p* < 0.01 based on *t*-tests. Underlying numerical data and statistical analysis for Fig 5D and 5E can be found in S1 Data.

An alternative model to explain the apparent discrepancy between the increased protein levels of EcR-USP and the decreased EcR target gene expression in *cdk8* or *cycC* mutants is that the CDK8-CycC complex is required for EcR-USP binding to the promoters of EcR target genes. In this model, the accumulated EcR-USP in nuclei may not effectively stimulate the target gene expression in the absence of CDK8 or CycC. To test this hypothesis, we used the antibodies against EcR or USP to ascertain protein localization on polytene chromosomes, which provide a straightforward method for rapid detection of the genome-wide localization of chromatin-binding proteins [77,78]. In polytene chromosome spreads from wild-type larvae, EcR (Fig 5A) and USP (Fig 5A’) antibodies stain distinct bands that largely overlap with each other. However, we could hardly detect any signal of anti-USP staining on polytene chromosome spreads from *cdk8* and *cycC* mutants that were prepared and imaged under the same conditions (Fig 5B’ and 5C’). Similarly, the signal of the anti-EcR antibody staining was significantly reduced on the polytene chromosome from the *cdk8* and *cycC* mutants (Fig 5B and 5C), compared to the control (Fig 5A). To validate the consequence of this reduction of EcR-USP binding to polytene chromosomes, we examined the expression of EcR-target genes in the *cdk8* and *cycC* mutant salivary glands at the L3 wandering stage using qRT-PCR. Similar to the data from whole-body analysis (Fig 2B), the levels of EcR activated genes were significantly reduced in *cdk8* and *cycC* mutant salivary glands than the control (Fig 5D). These observations suggest that the recruitment of EcR and USP to their target promoters is defective in *cdk8* and *cycC* mutants.

To further validate these observations, we performed chromatin immunoprecipitation (ChIP) assay to examine whether the presence of USP at EcR target gene promoters, such as *E74*, *E75*, *E78*, and *Hsp27*, was affected in *cdk8* and *cycC* mutant larvae. As shown in Fig 5E, the binding of USP to the promoters of these EcR-USP target genes was diminished in *cdk8* and *cycC* mutants. These data are consistent with the reduced binding of EcR and USP to the polytene spreads in *cdk8* and *cycC* mutants (Fig 5B and 5B’ and 5C and 5C’). Taken together, these observations suggest that the CDK8-CycC complex is required for the recruitment of EcR and USP to their target genes.

### Physical Interactions between CDK8 and EcR-USP

**Fig 6:**
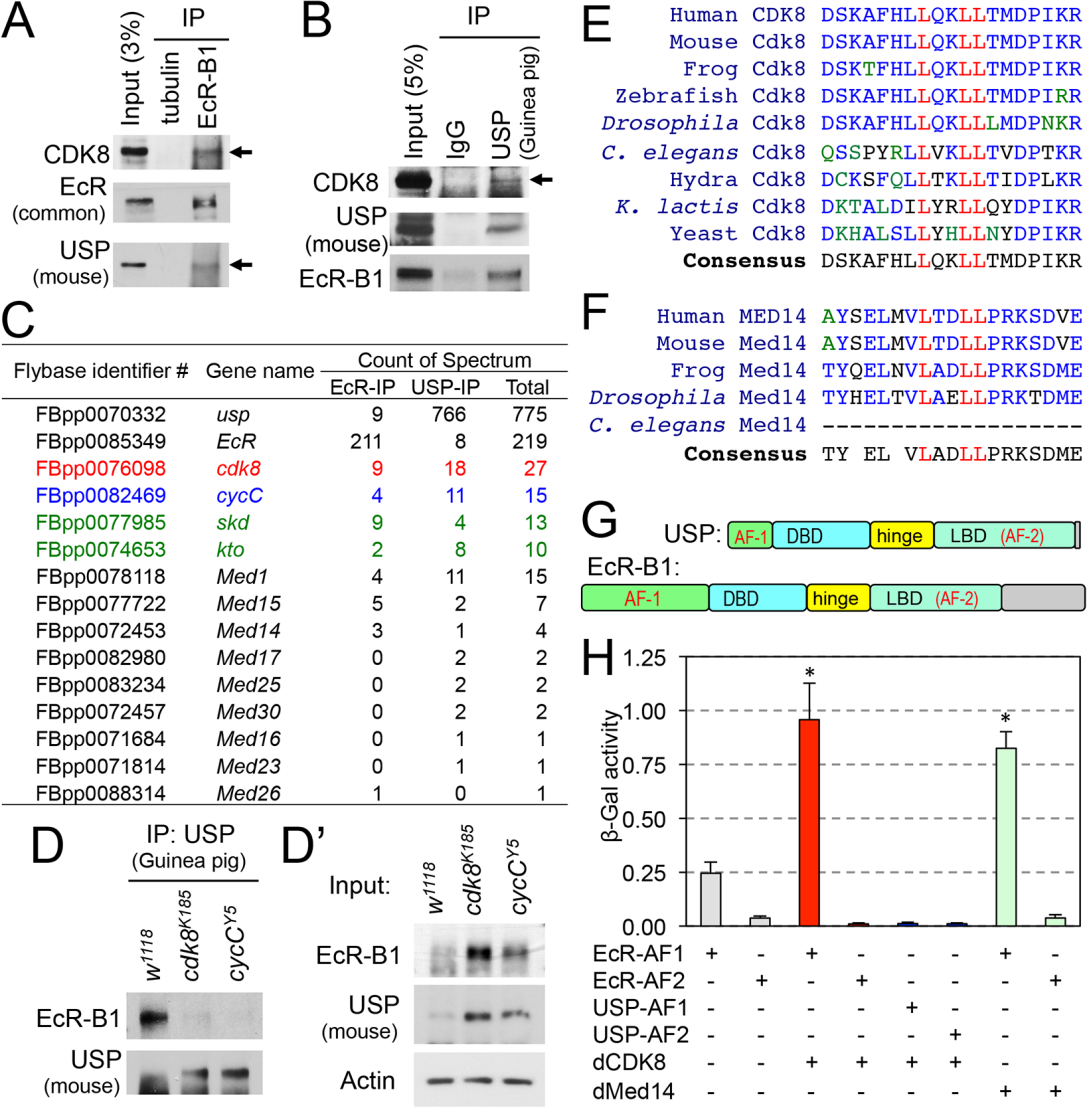
Biochemical interactions between CDK8-CycC and EcR-USP. (A) EcR-B1 co-immunoprecipitates with CDK8 in white prepupae; USP serves as the positive control. (B) USP co-immunoprecipitates with CDK8 in white prepupae; EcR serves as the positive control. (C) Mediator subunits that can co-immunoprecipitate with EcR or USP are identified by LC-MS/MS analysis. Subunits of the CDK8 submodule are shown in color. The results were combined from two biological replicates. (D) Immunoprecipitation of EcR by using anti-USP (guinea pig) antibody in white prepupae of *w* ^*1118*^, *cdk8* ^*K185*^, and *cycC* ^*Y5*^ mutants, and the input is shown in (D’). (E) CDK8 has a LXXLL motif that is highly conserved from yeast to human. The LXXLL motif is highlighted in red. (F) The LXXLL motif in Med14 is conserved from *Drosophila* to humans, but it is not present in *Caenorhabditis elegans*. (G) Schematic diagram of the EcR-B1 and USP protein depicting the two activating domains (AF1 and AF2), DNA-binding domain (DBD) and the ligand-binding domains (LBD). (H) Yeast two-hybrid analyses show that EcR-AF1, but not EcR-AF2 or USP-AF1/2, can directly bind to CDK8 and Med14. Underlying numerical data and statistical analysis for Fig 6H can be found in S1 Data.

To test the possibility that CDK8-CycC interacts with EcR or USP in vivo, we analyzed whether EcR or USP could co-immunoprecipitate with CDK8 in white prepupae. As shown in Fig 6A, EcR-B1 co-immunoprecipitated endogenous CDK8. Similarly, CDK8 was co-immunoprecipitated with USP (Fig 6B). These results suggest that CDK8 can interact with the EcR-USP complex in vivo. To test whether other Mediator subunits can co-immunoprecipitate with EcR and USP, we performed mass spectrometry analysis for proteins that immunoprecipitated with either EcR or USP in wild-type white prepupae. As shown in Fig 6C, multiple Mediator subunits, including the subunits of the CDK8 submodule, Med12 (encoded by *kohtalo* or *kto*), Med13 (encoded by *skuld* or *skd* [79–81]), CDK8, and CycC, co-immunoprecipitated with EcR and USP. This assay also identified several known cofactors for EcR-USP, such as Hsp70, Taiman, Smr, Rig, dDOR, and Utx (S2 Table). These results validated and significantly expanded our co-immunoprecipitation data (Fig 6A and 6B), suggesting that the Mediator complexes may function as transcriptional cofactors for EcR-USP.

To address whether the interaction between EcR and USP is affected by CDK8-CycC, we tested whether USP could co-immunoprecipitate EcR in *cdk8* and *cycC* mutants as efficient as control during the white prepupal stage. As expected, USP co-immunoprecipitated with EcR-B1 in the control; however, despite the elevated levels of EcR and USP in the mutants (Fig 6D’), much less EcR-B1 could be co-immunoprecipitated with USP in *cdk8* or *cycC* mutants (Fig 6D), consistent with the reduced USP binding to EcR targets in the mutants (Fig 5). This result suggests that CDK8-CycC normally functions to enhance the EcR-USP interaction, which is required for EcR-USP binding to the promoters of EcR target genes.

Many transcriptional cofactors of nuclear receptors are known to possess a conserved signature amino acid motif LXXLL (where L is leucine and X is any amino acid), as the interaction surfaces [65,66]. For example, via this LXXLL motif, the Mediator subunits (MED1 and MED14) and other cofactors interact with mammalian nuclear receptors, such as androgen receptor, estrogen receptor, glucocorticoid receptor, thyroid hormone receptor and RXR [65,66,82,83]. This LXXLL motif is also found in several transcription coactivators for EcR [13], such as Taiman, TRR, Rig, dDOR, dDEK, Hsp90, and Hsc70 [24–29,84]. Interestingly, we have found that CDK8, but not CycC, has a LXXLL motif that is highly conserved from yeasts to human (Fig 6E). The X-ray crystal structure of the human CDK8-CycC complex shows that this leucine-rich motif is localized on the surface of the CDK8 protein [52]. In addition, the two LXXLL motifs in MED1 are vertebrate-specific and they are not present in flies or worms (S8A Fig), while *Drosophila* Med14 has one LXXLL motif that is conserved from flies to humans but not in worms (Fig 6F).

To test whether EcR or USP may directly interact with CDK8 and Med14, we performed yeast two-hybrid assays. We focused on the EcR-B1 isoform, because *cdk8* and *cycC* mutants resemble *EcR-B1* mutants (Fig 1) and EcR-B1 is the major isoform that controls the larval–pupal transition [53]. Similar to other nuclear receptors, EcR and USP contain a ligand-independent activation function (AF1) domain at their N-termini, followed by a DNA-binding domain (DBD) and a ligand-binding domain (LBD) that contains the ligand-dependent activation function (AF2) (Fig 6G) [6,13]. We observed that EcR-AF1, but not EcR-AF2, could directly bind to CDK8 (Fig 6H). In contrast, CDK8 did not bind to either AF1 or AF2 of USP (Fig 6H). Similarly, EcR-AF1, but not EcR-AF2, directly interacted with the fragment of Med14 that contains the LXXLL motif (Fig 6H). One caveat of these analyses is that the ligand 20E is not present in this assay, thus it is possible that the ligand may be required for EcR-AF2 to interact with CDK8, Med14, or other Mediator subunits. Nevertheless, these data suggest that the Mediator complexes are involved in regulating EcR-dependent gene expression through direct interactions between EcR and CDK8 or Med14 (S8B Fig).

### Levels of CDK8-CycC, EcR-USP, and SREBP during the Larval–Pupal Transition

**Fig 7:**
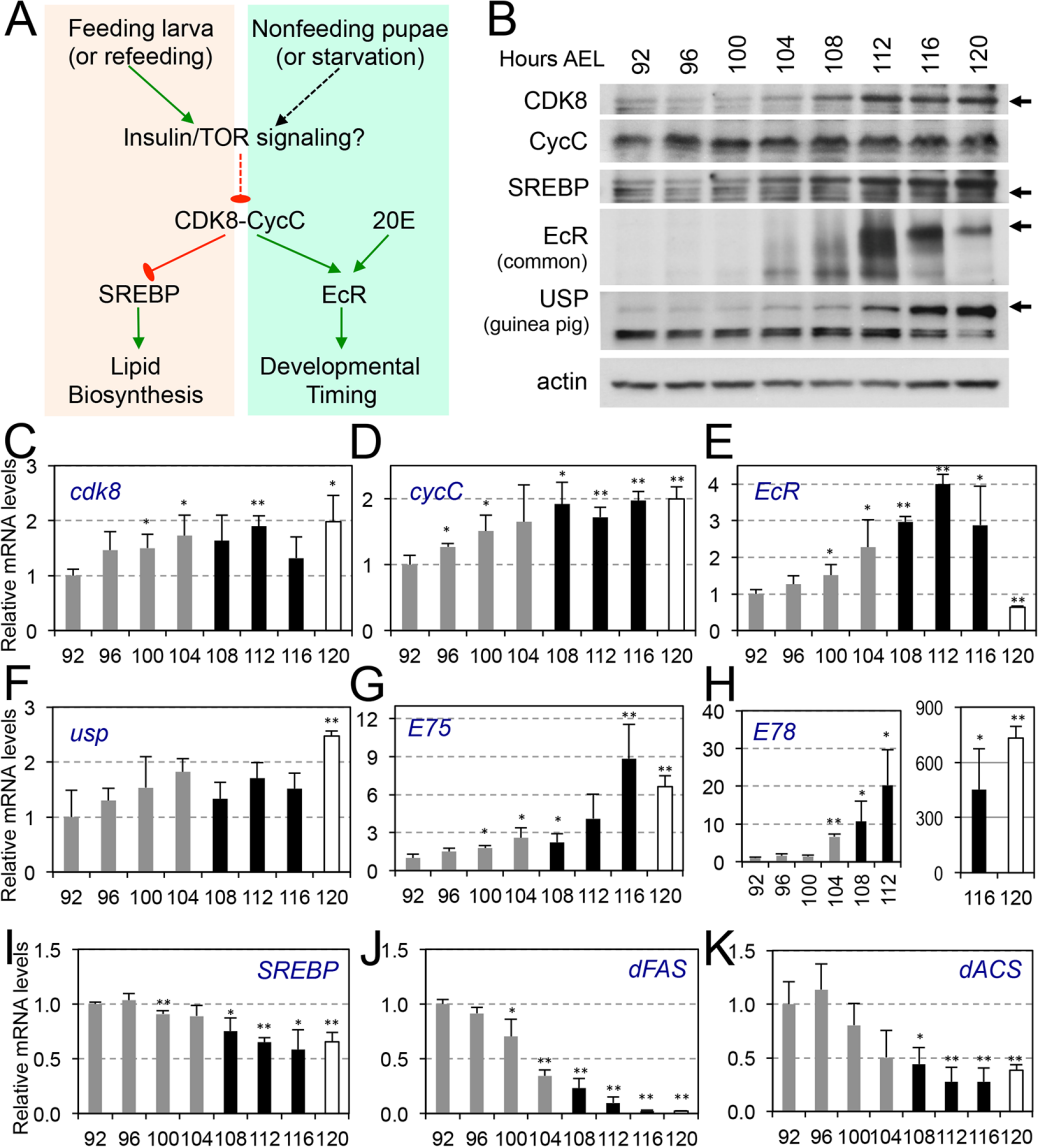
CDK8-CycC may couple nutrient intake, lipid biosynthesis, and developmental timing. (A) Model for the CDK8-SREBP/EcR regulatory network: In response to nutrient intake, CDK8-CycC may coordinately regulate lipogenesis by directly inhibiting SREBP-activated gene expression and developmental timing by activating EcR-activated gene expression during the larval–pupal transition. Arrows represent activation, and blunt arrows represent inhibition. (B) The protein levels of CDK8, CycC, SREBP, EcR-B1 and USP (upper band is the 54 kDa full-length USP) in wild-type larvae from L3 (92 hr AEL) to the WPP stage (120 hr AEL). For SREBP, the lower band (approximately 49 kDa, arrow) is the mature nuclear form, while the upper band (53–54 kDa) is the N-terminal fragment of SREBP after cleavage by the S1P (see S5B and S5C Fig for detailed analyses of these SREBP isoforms). (C–K) The mRNA levels of *cdk8*, *cycC*, *EcR*, *usp*, *E75*, *E78*, *SREBP*, *dFAS*, and *dACS* from L3 (92 hr AEL) to the WPP stage (120 hr AEL). The black bars represent the wandering stage, while the white bars represent the WPP stage. The *x*-axis represents the number of hours AEL. * *p* < 0.05; ** *p* < 0.01 based on *t*-tests. Underlying numerical data and statistical analysis for Fig 7C, 7D, 7E, 7F, 7G, 7H, 7I, 7J, and 7K can be found in S1 Data.

Recently, we have reported that CDK8-CycC plays a key role in regulating lipogenesis in *Drosophila* and mammals by directly inhibiting the transcriptional activity of SREBPs [46]. Since the wandering behavior triggered by a pulse of 20E may mark a fundamental transition in energy metabolism from SREBP-dependent lipogenesis in feeding larvae to lipolysis in nonfeeding pupae, our data showing that CDK8 regulates EcR- and SREBP-dependent transcription prompt us to hypothesize that CDK8-CycC may integrate feeding-stimulated lipogenesis and ecdysone-regulated metamorphosis during the larval–pupal transition (Fig 7A).

To assess the plausibility of this hypothesis, we first analyzed the protein levels of CDK8, CycC, SREBP, EcR and USP from mid-L3 larval stage (92 hr AEL) to WPP stage (120 hr AEL) by Western blot. In our experiments, the larvae started moving out of food approximately104 hr AEL, wandering stage occurred between 108 and 116 hr AEL, and then they reached WPP stage at approximately120 hr AEL. As shown in Fig 7B, the level of CDK8 is significantly increased during the wandering stage, which coincides with the abrupt increase of EcR and USP proteins. In contrast, the protein levels of CycC and SREBP were not significantly altered.

To test whether the levels of EcR-USP and SREBP correlate with the expression of their target genes, we analyzed the expression of their target genes using qRT-PCR. We observed that the mRNA levels of *cdk8* and *cycC* are gradually increased during L3 (Fig 7C and 7D), which is supported by our measurement of their levels from early L3 (84 hr AEL) to pupal stage (72hr APF) (S9A Fig). Although the expression of *usp* is not significantly increased, the mRNA levels of *EcR* and EcR-target genes, such as *E74*, *E75*, and *E78*, are significantly increased during the wandering stage (Figs 7E–7H and S9B). In contrast to *EcR* and EcR target genes, the mRNA levels of *SREBP*, and particularly SREBP-target genes, such as *dFAS*, *dACC* and *dACS*, are significantly decreased during the wandering and WPP stages (Figs 7I–7K and S9C). Importantly, the patterns of change for SREBP target genes and EcR target genes appear opposite, and the transition occurs during the wandering stage, suggesting that the onset of wandering stage may represent a turning point for the increase of CDK8 and EcR-USP but the opposite trend of SREBP activity during the late L3 stage. The wandering behavior is accompanied by the cessation of feeding, thus the wandering stage may mark the major shift from lipogenesis in feeding larvae to EcR-regulated pupariation. These changes are suggestive and correlative, thus we performed additional experiments to test the relationship between nutrient intake and activities of SREBP and EcR as described below.

### Starvation of the Feeding Larvae Lead to Precocious Increase of CDK8, EcR, and USP

**Fig 8:**
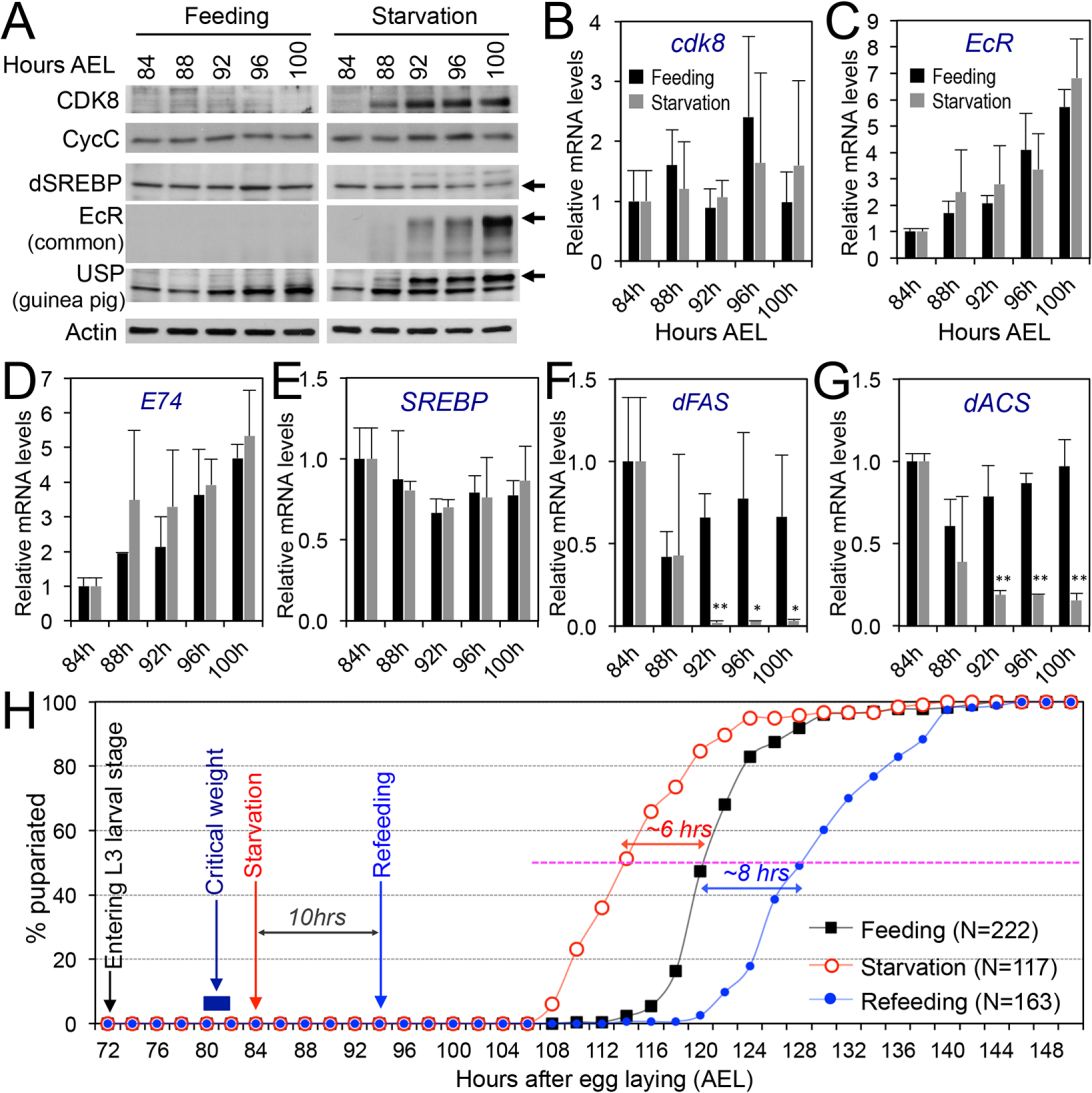
The effects of starvation on the CDK8-SREBP/EcR regulatory network and timing for the larval–pupal transition. (A) The protein levels of CDK8, CycC, mature SREBP (arrow), EcR-B1 (arrow), and USP (arrow, full-length USP) in feeding versus starved larvae from 84 hr to 100 hr AEL. (B–G) The mRNA levels of *cdk8*, *EcR*, *E74*, *SREBP*, *dFAS*, and *dACS* in feeding versus starved larvae from 84 hr to 100 hr AEL. The *x*-axis represents the number of hours AEL. * *p* < 0.05; ** *p* < 0.01 based on *t*-tests. (H) The effect of starvation and refeeding on the timing of the larval–pupal transition in wild-type (*w* ^*1118*^) larvae. The larvae enter into L3 at 72 hr AEL and reach critical weight between 80 and 82 hr AEL. The larvae were starved starting 84 hr AEL and the timing of pupariation was analyzed once every two hours. For the refeeding experiments, the larvae were put back on normal food after 10 hr of starvation (94 hr AEL). Underlying numerical data and statistical analysis for Fig 8B, 8C, 8D, 8E, 8F, 8G, and 8H can be found in S1 Data.

Because CDK8-CycC directly regulates the transcriptional activity of both SREBP and EcR-USP, we sought to examine whether CDK8-CycC was actively involved in coordinating lipogenesis and metamorphosis in response to changes in nutrient intake triggered by wandering behavior. Since starvation of feeding larvae prematurely turns off nutrient intake, we asked whether starvation of the feeding larvae could precociously regulate the CDK8-SREBP/EcR network outlined in Fig 7A. *Drosophila* larvae reach critical weight between 80 hr and 82 hr AEL, and continue feeding for about 20 hr before the onset of wandering stage [85]. Therefore, we starved larvae during the first half of the post-critical weight feeding stage (84–100 hr AEL), and then analyzed levels of CDK8, CycC, SREBP, EcR, and USP by Western blot. As shown in Fig 8A, the levels of CDK8, EcR-B1, and the full-length USP are barely detectable in normal feeding larvae during 84–100 hr AEL, but all of them are significantly increased after 4–8 hr of starvation (88 or 92 hr AEL). In contrast, the level of nuclear SREBP was decreased after 12 hr of starvation (96 hr AEL), while CycC was not significantly affected by starvation (Fig 8A). These data show that starvation indeed leads to precocious reduction of mature form of SREBP and up-regulation of CDK8, EcR, and USP. Interestingly, the mRNA levels of these factors are not significantly affected by starvation (Figs 8B, 8C and 8E, S10A and S10B), suggesting post-transcriptional regulation of CDK8, EcR, USP, and SREBP by starvation.

Next, we examined whether the expression of EcR and SREBP target genes was affected by starvation using qRT-PCR. Consistent to reduced level of mature SREBP protein and increased CDK8, SREBP target genes such as *dFAS* and *dACS* are strongly reduced after 8 hr of starvation (Figs 8F and 8G). Although EcR and USP levels are significantly increased after starvation, expression of EcR target genes, such as *E74*, *E75* and *E78*, is not significantly affected by starvation (Figs 8D, S10C and S10D), suggesting that increase of EcR-USP alone is not sufficient to induce EcR target gene expression. To test whether the ecdysone biosynthesis is affected by starvation, we measured ecdysteroid titer and found no significant effect of starvation on ecdysone biosynthesis during the 84–100 hr AEL (S10E Fig). Although it is unclear whether ecdysone biosynthesis is accelerated by starvation between 100 and 120 hr AEL (see below, Fig 8H), this observation (S10E Fig) may explain why EcR target genes are not induced by elevated EcR-USP in starved larvae during the 16-hr period that we analyzed. Together, these results suggest that starvation precociously up-regulates CDK8-CycC and EcR-USP, but down-regulates SREBP and SREBP activity, all post-transcriptionally.

Furthermore, we analyzed the effect of starvation on the timing of the larval–pupal transition. We observed that starvation of the wild-type larvae after they reached critical weight led to approximately 6 hr earlier onset of pupariation (Fig 8H, red line) and formation of smaller pupae than control (S10F Fig). These observations are consistent to the predicted effects on the CDK8-EcR/SREBP network when nutrient intake is stopped early by starvation (Fig 7A).

### Refeeding of the Starved Larvae Reduces the Levels of CDK8, EcR, and USP

**Fig 9:**
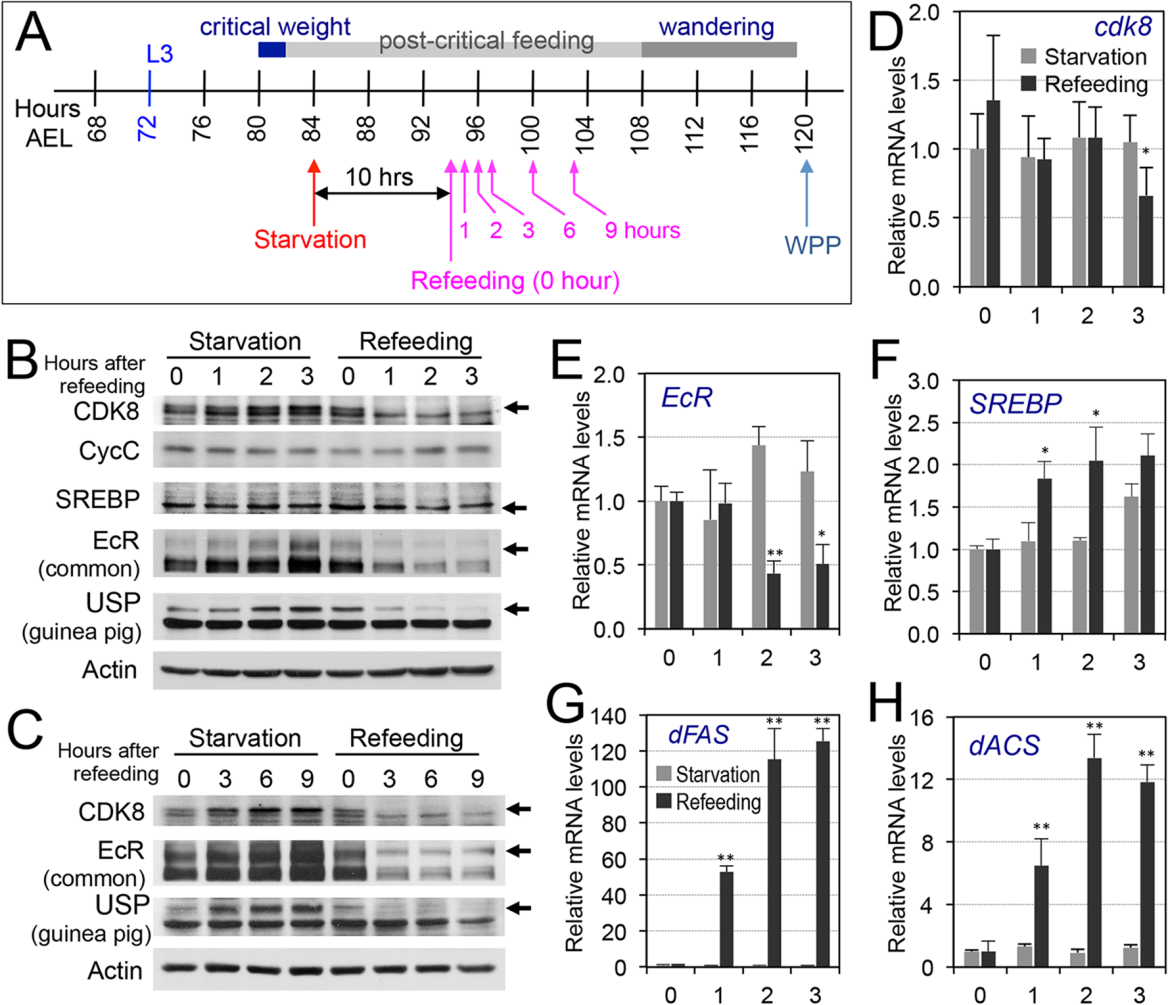
The effects of refeeding of starved larvae on the CDK8-SREBP/EcR regulatory network. (A) The experimental scheme for the refeeding treatment. Briefly, the wild-type larvae were starved for 10 hr (from 84 hr AEL to 94 hr AEL), and they were then transferred back onto normal food. Samples were collected after 1, 2, 3, 6, 9 hr after refeeding for further analyses. (B) The protein levels of CDK8, CycC, SREBP, EcR-B1 and USP in starved versus refed larvae after 1, 2, 3 hr of refeeding. (C) The protein levels of CDK8, EcR-B1, and USP in starved versus refed larvae after 3, 6, 9 hr of refeeding. The control (anti-actin) is the same as the S12A Fig (D–H) The mRNA levels of *cdk8*, *EcR*, *SREBP*, *dFAS*, and *dACS* in starved versus refed larvae after 1, 2, 3 hr of refeeding. The *x*-axis represents the number of hours for refeeding. * *p* < 0.05; ** *p* < 0.01 based on *t*-tests. Underlying numerical data and statistical analysis for Fig 9D, 9E, 9F, 9G, and 9H can be found in S1 Data.

Previously, we reported that refeeding of the starved larvae strongly activated the expression of lipogenic genes such as *dFAS*, while over-expression of CycC in fat body significantly hampered the refeeding-induced *dFAS* expression [46]. Therefore, to further analyze the effect of nutrition and feeding on the CDK8-EcR-SREBP network, we tested whether refeeding of starved larvae could have opposite effects on the CDK8-EcR/SREBP regulatory network to starvation. Specifically, we starved wild-type larvae at 84 hr AEL for 10 hr, and then collected the refeeding larvae after they were transferred back to normal food for 0, 1, 2, 3, 6, or 9 hr (Fig 8H, blue line; Fig 9A). We observed that refeeding for 1 to 3 hr potently reduced the protein levels of CDK8, EcR and USP (Fig 9B). Except *EcR*, the mRNA levels of *cdk8* and *usp* are not obviously affected by refeeding (Figs 9D and 9E, and S11B), suggesting a post-transcriptional regulation of these factors by refeeding. Similar observations were made after refeeding for 6 or 9 hr (Fig 9C and S12). Importantly, these changes are opposite to the effect of starvation (Fig 8A), supporting the inhibitory effects of feeding or refeeding on CDK8 (Fig 7A). Although both EcR and USP levels are reduced in refed larvae, expression of EcR-target genes are not obviously affected (S11C–S11E Fig and S12F and S12G Fig). Perhaps, biosynthesis of 20E or other cofactors for EcR-USP dependent transcription are not present in refed larvae in the time window that we analyzed. Indeed, the refed larvae could pupariate, but with approximately 8 hr of delay (Figs 8H and S10F). In addition, we did not observe any obvious changes in the level of mature SREBP proteins, but expression of *SREBP* and the SREBP target genes were significantly increased in refed larvae (Figs 9F–9H and S12H–S12K), suggesting a potent stimulatory effect of refeeding on SREBP activity. Taken together, these results are largely consistent with the model that CDK8-CycC links the nutrition intake to EcR-USP and the activity of SREBP, suggesting that CDK8-CycC functions as a signaling node for coordinating lipid homeostasis and developmental timing in response to nutrient cues (Fig 7A).

## Discussion

Through EcR-USP, ecdysone plays pivotal roles in controlling developmental timing in *Drosophila*. In this study, we show that *cdk8* or *cycC* mutants resemble *EcR-B1* mutants and CDK8-CycC is required for proper activation of EcR-target genes. Our molecular and biochemical analyses suggest that CDK8-CycC and the Mediator complexes are directly involved in EcR-dependent gene activation. In addition, the protein levels of CDK8 and CycC are up-regulated at the onset of the wandering stage, closely correlated with the activation of EcR-USP and down-regulation of SREBP-dependent lipogenesis during the larval–pupal transition. Remarkably, starvation of the feeding larvae leads to premature up-regulation of CDK8 and EcR-USP, and precocious down-regulation of SREBP, while refeeding of the starved larvae results in opposite effects on the CDK8-SREBP/EcR network. Thus, we propose that CDK8-CycC serves as a key mediator linking food consumption and nutrient intake to EcR-dependent developmental timing and SREBP-dependent lipogenesis during the larval–pupal transition.

### CDK8-CycC As a Transcription Cofactor for EcR-USP

The Mediator complex is composed of up to 30 different subunits, and biochemical analyses of the Mediator have identified the small Mediator complex and the large Mediator complex, with the CDK8 submodule being the major difference between the two complexes [38,39,86]. Several reports link EcR and certain subunits of the Mediator complex. For example, Med12 and Med24 were shown to be required for ecdysone-triggered apoptosis in *Drosophila* salivary glands [87–89]. It was recently reported that ecdysone and multiple Mediator subunits could regulate cell-cycle exit in neuronal stem cells by changing energy metabolism in *Drosophila*, and specifically, EcR was shown to co-immunoprecipitate with Med27 [90]. However, exactly how Mediator complexes are involved in regulating EcR-dependent transcription remains unknown. Our data suggest that CDK8 and CycC are required for EcR-activated gene expression. Loss of either CDK8 or CycC reduced USP binding to EcR target promoters, diminished EcR target gene expression, and delayed developmental transition, which are reminiscent of *EcR-B1* mutants [53]. Importantly, our mass spectrometry analysis for proteins that co-immunoprecipitate with EcR or USP has identified multiple Mediator subunits, including all four subunits of the CDK8 submodule. Taken together, previous works and our present work highlight a critical role of the Mediator complexes including CDK8-CycC in regulating EcR-dependent transcription.

How does CDK8-CycC regulate EcR-activated gene expression? Our biochemical analyses show that CDK8 can interact with EcR and USP in vivo and that CDK8 can directly interact with EcR-AF1. These observations, together with the current understanding of how nuclear receptors and Mediator coordinately regulate transcription, suggest that CDK8-CycC may positively and directly regulate EcR-dependent transcription (S8B Fig). Our yeast two-hybrid analysis indicates that the recruitment of CDK8-CycC to EcR-USP can occur via interactions between CDK8 and the AF1 domain of EcR. Interestingly, this assay also revealed a direct interaction between EcR-AF1 and a fragment of Med14 that contains the LXXLL motif. In future work, it will be interesting to determine whether CDK8 and Med14 compete with each other in binding with the EcR-AF1, whether they interact with EcR-AF1 sequentially in activating EcR-dependent transcription, and how the Mediator complexes coordinate with other known EcR cofactors in regulating EcR-dependent gene expression.

In *cdk8* or *cycC* mutants, the binding of USP to the promoters of the EcR target genes is significantly compromised, even though nuclear protein levels of both EcR and USP are increased. It is unclear how CDK8-CycC positively regulates EcR-USP binding to EcREs near promoters. CDK8 can directly phosphorylate a number of transcription factors, such as Notch intracellular domain, E2F1, SMADs, SREBP, STAT1, and p53 [42,43,46]. Interestingly, the endogenous EcR and USP are phosphorylated at multiple serine residues, and treatment with 20E enhances the phosphorylation of USP [70,91,92]. Protein kinase C has also been proposed to phosphorylate USP [93,94]. It will be interesting to determine whether CDK8 can also directly phosphorylate either EcR or USP, thereby potentiating expression of EcR target genes and integrating signals from multiple signaling pathways.

### Potential Roles of CDK8-CycC in Regulating the Biosynthesis of Ecdysone

Although we favor a direct role for CDK8-CycC to regulate EcR-USP activated gene expression, we could not exclude the potential contribution of impaired biosynthesis of 20E to the developmental defects in *cdk8* or *cycC* mutants. For example, the expression of genes involved in synthesis of 20E, such as *sad* and *spok*, is significantly reduced in *cdk8* or *cycC* mutant larvae. Indeed, the ecdysteroid titer is significantly lower in *cdk8* mutants than control from the early L3 to the WPP stages, and feeding the *cdk8* mutant larvae with 20E can partially reduce the retardation in pupariation. Nevertheless, impaired ecdysone biosynthesis alone cannot explain developmental defects that we characterized in this report for the following reasons. First, feeding *cdk8* or *cycC* mutants with 20E cannot rescue the defects in pupal morphology, developmental delay, and the onset of pupariation. Second, the expression of *EcRE-lacZ* reporter in *cdk8* or *cycC* mutant salivary glands cannot be as effectively stimulated by 20E treatment as in control. Third, knocking down of either *cdk8* or *cycC* in PG did not lead to obvious defects in developmental timing. Therefore, the most likely scenario is that the *cdk8* or *cycC* mutants are impaired not only in 20E biosynthesis in the PG, but also in EcR-activated gene expression in peripheral tissues. Defects in either ecdysone biosynthesis or EcR transcriptional activity will generate the same outcome: diminished expression of the EcR target genes, thereby delayed onset of pupariation.

How CDK8-CycC regulates biosynthesis of ecdysone in PG remains unknown. Several signaling pathways have been proposed to regulate ecdysone biosynthesis in *Drosophila* PG, including PTTH and *Drosophila* insulin-like peptides (dILPs)-activated receptor tyrosine kinase pathway and Activins/TGFβ signaling pathway [95,96]. Interestingly, CDK8 has been reported to regulate the transcriptional activity of SMADs, transcription factors downstream of the TGFβ signaling pathway, in both *Drosophila* and mammalian cells [97,98]. Thus, it is conceivable that the effect of *cdk8* or *cycC* mutation on ecdysone biosynthesis may due to dysregulated TGFβ signaling in the PG.

### Effects of Starvation and Refeeding on CDK8

Our effort to explore the potential role of food consumption and nutrient intake on CDK8-CycC has resulted an unexpected observation that the protein level of CDK8 is strongly influenced by starvation and refeeding: starvation potently increased CDK8 level, while refeeding has opposite effect, and both occur post-transcriptionally (Figs 8 and 9). The importance of this observation is highlighted in two aspects. First, considering the generally repressive role of CDK8 on Pol II-dependent gene expression, up-regulation of CDK8 may provide an efficient way to quickly tune down most of the Pol II-dependent transcription in response to starvation; while down-regulation of CDK8 in response to refeeding may allow many genes to express when nutrients are abundant. Second, it will be necessary to test whether the effects of nutrient intake on CDK8-CycC is conserved in mammals. If so, considering that both CDK8 and CycC are dysregulated in a variety of human cancers [43], the effects of nutrient intake on CDK8 may have important implications in not only our understanding of the effects of nutrients on tumorigenesis, but also providing nutritional guidance for patients with cancer.

Major dietary components including carbohydrates, lipids, and proteins, can strongly influence the developmental timing in *Drosophila* [2]. Excessive dietary carbohydrates repress growth and potently retard the onset of pupariation [99–101]. One elegant model proposed to explain how high sugar diet delays developmental timing is that high sugar diet reduces the activity of the Target of Rapamycin (TOR) in the PG, thereby reducing the secretion of ecdysone and delaying the developmental transition [102]. Previously, we reported that insulin signaling could down-regulate CDK8-CycC, and that ectopic expression of CycC could antagonize the effect of insulin stimulation in mammalian cells, as well as the effect of refeeding on the expression of *dFAS* in *Drosophila* [46]. Although the mRNA levels of TOR and insulin receptor (InR) are not significantly affected in *cdk8* or *cycC* mutants (Fig 3B), it is necessary to further study whether and how different dietary components may regulate CDK8-CycC in the future.

### CDK8-CycC Links Fat Metabolism and Developmental Timing with Nutritional Cues

Our developmental genetic analyses of the *cdk8* and *cycC* mutants have revealed major defects in fat metabolism and developmental timing ([46]; this work). De novo lipogenesis, which is stimulated by insulin signaling, contributes significantly to the rapid increase of body mass during the constant feeding larval stage. This process is terminated by pulses of ecdysone that trigger the wandering behavior at the end of the L3 stage, followed by the onset of the pupariation. Insulin and ecdysone signaling are known to antagonize each other, and together determine body size of *Drosophila*. The genetic interaction is established, but the detailed molecular mechanisms are not [1,25,103,104]. The SREBP family of transcription factors controls the expression of lipogenic enzymes in metazoans and the expression of cholesterogenic enzymes in vertebrates [105,106]. Our previous work shows that CDK8 directly phosphorylates the nuclear SREBP proteins on a conserved threonine residue and promotes the degradation of nuclear SREBP proteins [46]. Consistent with the lipogenic role of SREBP and the inhibitory role of insulin to CDK8-CycC [46], the transcriptional activity of SREBP is high while the levels of CDK8-CycC and EcR-USP are low prior to the onset of wandering stage. Subsequently during the wandering and non-mobile, non-feeding pupal stage, the transcriptional activity of SREBP is dramatically reduced, accompanied by the significant accumulation of CDK8-CycC and EcR-USP (Fig 7).

The causal relationship of these phenomena was further tested by our starvation and refeeding experiments. On the one hand, we observed that the levels of CDK8, EcR and USP are potently induced by starvation, while the mature SREBP level and the transcriptional activity of SREBP are reduced by starvation (Fig 8). Starvation of larvae prior to the two nutritional checkpoints in early L3, known as minimum viable weight and critical weight, which are reached almost simultaneously in *Drosophila*, will lead to larval lethality; while starvation after larvae reach the critical weight will lead to early onset of pupariation and formation of small pupae [9,85,107,108]. Thus, this nutritional checkpoint ensures the larvae have accumulated sufficient growth before metamorphosis initiation [2,85]. If we regard the status with high CDK8, EcR, and USP as an older or later stage, these results indicate that starvation shifts the regulatory network precociously, which is consistent with the regulatory network outlined in Fig 7A and the observed premature pupariation (Fig 8H). On the other hand, our analyses of refed larvae show that refeeding potently reduced the levels of CDK8, EcR and USP (Fig 9). If we consider the status with low CDK8, EcR, and USP as a younger or earlier stage, these results indicate that refeeding delays the activation of this network, which is consistent with our model (Fig 7A) and delayed pupariation as observed (Fig 8H). Taken together, our results based on starved and refed larvae suggest that CDK8-CycC is a key regulatory node linking nutritional cues with de novo lipogenesis and developmental timing (Fig 7A).

The larval–pupal transition is complex and dynamic. Although the expression of SREBP target genes fit well with the predicted effects of starvation and refeeding, the expression of EcR targets during the stage that we analyzed does not reflect the changes in the protein levels of EcR and USP (Figs 8 and 9). It is reasonable to consider that CDK8-CycC and EcR-USP are necessary, but not sufficient, for the activation of EcR target genes. One possibility is that there is a delay on synthesis of 20E or other cofactors that are required for EcR-activated gene expression in response to starvation. Indeed, we measured the 20E levels during the first 16 hr of starvation and observed no significant difference between fed and starved larvae (S10E Fig). It will be necessary to further analyze the effect of starvation on 20E synthesis at later time points in the future.

Taken together, we propose a model whereby CDK8-CycC functions as a regulatory node that coordinates de novo lipogenesis during larval stage and EcR-dependent pupariation in response to nutritional cues (Fig 7A). It is likely that pulses of 20E synthesized in the PG, and subsequent behavioral change from feeding to wandering, ultimately trigger the transition from SREBP-dependent lipogenesis to EcR-dependent pupariation. The opposite effects of CDK8-CycC on SREBP- and EcR-dependent gene expression suggest that the role of CDK8 on transcription is context-dependent.

In conclusion, our study illustrates how CDK8-CycC regulates EcR-USP-dependent gene expression, and our results suggest that CDK8-CycC may function as a regulatory node linking fat metabolism and developmental timing with nutritional cues during *Drosophila* development.

## Materials and Methods

### *Drosophila* Stocks and Genetics

The null alleles of *cdk8* (*cdk8* ^*K185*^) and *cycC* (*cycC* ^*Y5*^) strains were provided by Drs. Muriel Boube and Henri-Marc Bourbon [50]. The *EcRE-lacZ* reporter and *ubi-Gal4* lines were obtained from Dr. Keith Maggert. The *P[hs-usp]* transgenic line [72,73] was obtained from the Bloomington *Drosophila* stock center. Embryos from *cycC* ^*Y5*^ germline clones were generated using the Flipase recombinase-mediated dominant female sterile technique [109]. All flies were maintained on standard cornmeal-molasses-yeast medium at 25°C.

### Antibodies

The anti-USP monoclonal antibody was provided by Dr. Rosa Barrio Olano. The anti-CycC polyclonal antiserum (peptide antibody in rabbits) was provided by Dr. Terry Orr-Weaver. Anti-EcR common (DDA2.7) and anti-EcR-B1 (AD4.4) monoclonal antibodies were obtained from Developmental Studies Hybridoma Bank, and the anti-actin (MA5-11869) monoclonal antibody was purchased from Thermo Scientific (Rockford, IL). The anti-CDK8 polyclonal antibody was generated by immunizing rabbits using peptide AA355~372 (KREFLTDDDQEDKSDNKR) as the antigen, anti-SREBP polyclonal antibody was generated using peptide AA360~378 (KDLLQLGTRPGRASKKRRE) as the antigen, and both were performed by Thermo Scientific. The antisera were purified by GST-CDK8 (AA1~372) or GST-SREBP (AA1~451) fusion proteins, respectively, using the protocol as described previously [110]. The anti-USP polyclonal antibody was generated by immunizing guinea pigs with GST-USP (full length) as the antigen, performed by Covance Research Products (Denver, PA). These fusion proteins were generated using the protocol described previously [111].

### Generation of the Rescue Strains

We generated the tagged genomic *cdk8* or *cycC* (approximately 7.5-kb) rescue constructs using backbone of the pVALIUM20 vector, which can be used for site-specific insertion with the PhiC31 integrase system [112]. For subcloning, we first linearized the pVALIUM20-gypsy-MSC10 vector by EcoRI (NEB). The gDNA segments for *cdk8* and *cycC* were PCR amplified from bacterial artificial chromosome (BAC) clones (CH322-104A8 for *cdk8* locus and CH321-46N21 for *cycC* locus) from the BACPAC Resources Center (http://bacpac.chori.org/home.htm). To ensure the fidelity of these PCR reactions, we used a high-fidelity DNA polymerase PrimeSTAR Max (Takara, Cat# R045A) and then purified all segments by gel extraction (QIAEX II). To join four DNA segments (pVALIUM20 backbone, two gDNA segments and one EGFP segment) seamlessly in a single reaction, we used In-Fusion HD system developed by Clontech (639649). This system requires that the sense and antisense PCR primers contain a 15bp overlap with the neighboring segment and 20–30bp segment specific sequence.

The primers with the 15bp overlapping sequence underlined are listed below: Cdk8 IN-1L: 5′-GTGGCTAGCAGAATTCAGGCACCCATTGGCGATG; Cdk8 IN-2: 5′-GTTGAAGCGCTGGAAGTTCTGCT; Cdk8 IN-3(EGFP): 5′-TTCCAGCGCTTCAACATGGTGAGCAAGGGCGAGGAG; Cdk8 IN-4(EGFP): 5′-TGTATCAGTCTCTCACTTGTACAGCTCGTCCATGCCG; Cdk8 IN-5: 5′- TGAGAGACTGATACATGCAGCATTTTTTC; Cdk8 IN-6LL: 5′- GGCTCTAGATGAATTATGCTCGCTGATTCCACGATCAG; CycC IN-1L: 5′- GTGGCTAGCAGAATTTCCTTCGAGGATCGCACCTG; CycC IN-2: 5′-ACGCTGAGGCGGTGGTTTC; CycC IN-3(EGFP-ATG): 5′-ATGCCACCGCCTCAGCGTGTGAGCAAGGGCGAGGAGCTG; CycC IN-4(EGFP): 5′-TATGAAGCTCTTCTACTTGTACAGCTCGTCCATGCCG; CycC IN-5: 5′-TAGAAGAGCTTCATAATCATTCATCATTAGC; and CycC IN-6L: 5′-GGCTCTAGATGAATTTGCTGGACCTATACAGACGCACG.

For the In-Fusion reaction, 100 ng of enzyme-digested, gel-purified vector were mixed with the PCR amplified segments at a molar ratio of 1 vector to 2 of each DNA segment in a total of 10 μl system buffered by In-Fusion HD Enzyme premix and the subsequent steps were carried out following the manufacturer’s instructions. The positive clones were selected and characterized by restriction enzyme digestion and sequencing. The rescue constructs were inserted into the second chromosome (attP40 site at 25C6) with the service provided by Genetic Services, Inc. This design facilitates genetic recombination since the endogenous *cdk8* and *cycC* genes are on the third chromosome.

### Microarray Analyses

The microarray analyses were described previously [46], and the data sets can be found in the ArrayExpress database (http://www.ebi.ac.uk/arrayexpress/; accession number E-MTAB-1066).

### Analysis of the mRNA Levels by Quantitative Reverse-Transcription-PCR (qRT-PCR) Assay

The RNA isolation, reverse transcription, the qRT-PCR analyses, and primers for the lipogenic enzymes were performed as described previously [46]. The primers used in the qRT-PCR assay are listed in S3 Table, and *Rp49* gene was used as the control. Primers for *InR*, *kni*, *mld*, *nvd*, *tor*, and *vvl* are adapted from [61].

### Ecdysteroid Measurements

Quantification of ecdysteroids in whole larvae was performed as described by [61,113] with the following modifications. Briefly, animals were homogenized in 0.25 ml 75% methanol, and then the supernatants were collected following centrifugation at 14,000 g for 15 min. The pellets were re-extracted in 0.1 ml methanol. The supernatants were combined, evaporated using a SpeedVac, and then re-dissolved in 0.5 ml ELISA buffer (Cayman Chemical). Ecdysteroids were measured using a commercial ELISA kit (Cayman Chemical) that detects 20E equivalents. Standard curves were generated using 20E (Cayman Chemical), and absorbance was measured at 405 nm on a microplate photometer (Thermo Scientific).

### 20E Rescue Experiments

The rescue experiments were performed as described previously [63]. Briefly, *cdk8* ^*K185*^ and *cycC* ^*Y5*^ homozygous mutants at late L2 and early L3 larvae were collected and placed in groups of 10 individuals in new vials containing food with 20E (Alexis or Cayman Chemical) ranging from 2.0 μM to 2.0 mM (Figs 3 and S4), and *w* ^*1118*^ larvae were treated in parallel as the control. Pupae were collected and photographed under a microscope.

### β-galactosidase Staining

The *EcRE-lacZ* reporter line was recombined with *cdk8* ^*K185*^ or *cycC* ^*Y5*^ mutant to generate the following genotypes: “*w* ^*1118*^; *EcRE-lacZ*; *cdk8* ^*K185*^ */TM6B*”, “*w* ^*1118*^; *EcRE-lacZ*; *cycC* ^*Y5*^ */TM6B*”. The “*w* ^*1118*^; *EcRE-lacZ*; *+*” line was used as the control. To ensure that we compare the salivary glands that are at the same developmental stage, we dissected the salivary gland from mid-L3 homozygous larvae (non-TM6B) of all these genotypes, and separated into two halves in Grace’s insect medium (HiMedia). One half was treated in Grace’s medium with 1μM 20E, while the other half from the same larva was cultured in Grace’s insect medium as the control. After 2.5 hr incubation at 25 ˚C, salivary glands were stained with X-gal solution (3.0 mM K_4_[Fe(CN)_6_], 3.0 mM K_3_[Fe(CN)_6_] in PBS with X-gal stock solution (8% in DMSO) added to a final concentration of 0.2%) at 37°C for 1 hr in the dark. The stained salivary glands were then transferred into 80% glycerol in PBS, mounted and photographed with a Leica DM2500 microscope.

### Immunostaining of Salivary Glands and Polytene Chromosomes

The salivary glands from the third-instar wandering larvae were dissected in PBS (phosphate buffered saline, pH7.4) and fixed in 5% formaldehyde in PBS for 10 min. After washing in PBT (PBS with 0.2% Triton X-100, pH7.4) for 4 times for 1 hour, the salivary glands were blocked in PBTB (0.2% BSA, 5% normal goat serum in PBT) for 1 hour at room temperature. The glands were then incubated with the anti-EcR-common DDA2.7 antibody (1:100) and anti-USP antibody (1:2,000, polyclonal antibody from guinea pigs) at 4 ˚C overnight on a nutator. After rinsing with PBT for 4 times, the glands were incubated with secondary antibodies (BODIPY-conjugated goat anti-mouse antibody 1:500 in PBTB; Alex594-conjugated goat anti-guinea pig antibody, 1:2,500 in PBTB) at room temperature for 2 hr. After standard nuclear counterstaining with DAPI (4′,6-Diamidino-2-phenylindole dihydrochloride, Sigma), the salivary glands were mounted on slides with Vectashield mounting media (Vector lab). For immunostaining of polytene chromosome, we followed the protocol described previously [114], and the following antibodies were used: anti-EcR-common DDA2.7 (1:200 in PBTB), anti-USP (Guinea pig, 1:200), BODIPY-conjugated goat anti-mouse antibody (1:500), and Alex594-conjugated goat anti-guinea pig antibody (1:1,000). Confocal images were taken with a Nikon Ti Eclipse microscope, and images were processed by Adobe Photoshop CS6 software.

### Western Blot Analysis

We separated the cytoplasmic, nuclear soluble, and nuclear insoluble fractions of protein extractions by following the protocol as described [115]. Western blot analysis was performed as previously described with minor modifications [46]. For whole cell extracts, homogenized larvae or pupae were lysed in a buffer containing 50 mM Tris HCl (pH 8.0), 0.1 mM EDTA, 420 mM NaCl, 0.5% NP-40, 10% glycerol, 1 mM dithiothreitol (DTT), 2.5 mM phenylmethanesulfonylfluoride (PMSF), protease and phosphatase inhibitors (the cOmplete Protease Inhibitor Cocktails and PhosSTOP, Roche Applied Science). Supernatants were collected after centrifugation at 2,000 *g* for 15 min at 4°C. Protein concentrations were measured with a Bradford protein assay kit (Bio-Rad). A given amount of whole cell extract was mixed with 4x Laemmli sample buffer (Bio-Rad). After boiling for 5 min, the proteins were resolved by 8% SDS-PAGE gel and transferred to PVDF membrane. The following antibodies were used: anti-EcR-common DDA2.7 (1:250), anti-USP (guinea pig, 1:2,000), anti-USP (monoclonal antibody, 1:1,000), anti-CDK8 (polyclonal antibody from rabbit, 1:50), anti-CycC (rabbit polyclonal antibody, 1:2,000), anti-dSREBP (rabbit polyclonal antibody, 1:100), and anti-actin monoclonal antibody (1:4,000, Thermo Scientific). The membranes were incubated with the corresponding HRP-conjugated secondary antibodies (1:2,500–1:10,000, Jackson ImmunoResearch) for 1 hr at room temperature. After washing, the HRP signals were visualized by the Western Lightening Plus ECL (PerkinElmer) according to the manufacturer’s instructions.

### The Co-immunoprecipitation (Co-IP) Assay

The co-IP assay was performed as described previously with minor modifications [116]. Briefly, the IP complex was prepared with 35 μL Magnetic Protein G beads (28-9670-66, GE Healthcare Life Sciences) and 5 μg primary antibody or IgG in 500 μL PBS and put on the rotator for 12–16 hr at 4°C. After incubation, the IP complex was washed with PBS twice and eventually removed all PBS. Lysates of 30 white prepupae per sample were prepared in the lysis buffer (150 mM NaCl, 50 mM Tris pH 8.0, 5 mM EDTA, 5 mM DTT, 0.1 mM PMSF, 0.5% NP-40, 2 mM Na_3_VO_4_, and protease inhibitor from Roche Applied Science). 400 μL of lysates were pre-cleared with 20 μL Magnetic Protein G beads on a rotator for 1 hr at 4°C, then the beads were discarded, and the lysates were mixed with IP complex and put on the rotator for 12–16 hr at 4°C. The IP complex was washed with lysis buffer (without protease inhibitor) five times, added 60 μL 2X sample buffer, denatured for 3 min at 95°C, and further analyzed by Western blot.

### Liquid Chromatography-Tandem Mass Spectrometry (LC-MS/MS) and Data Analysis

Each IP sample was run as a gel plug and proteins in the gel plug were reduced, carboxymethylated, digested with trypsin using standard protocols. Peptides were solubilized in 0.1% trifluoroacetic acid, and analyzed by Nano LC-MS/MS (Dionex Ultimate 3000 RLSCnano System interfaced with a Velos-LTQ-Orbitrap (ThermoFisher, San Jose, CA). Sample was loaded onto a self-packed 100 μm x 2 cm trap (Magic C18AQ, 5 μm 200 Å, Michrom Bioresources, Inc.) and washed with Buffer A (0.2% formic acid) for 5 min with a flow rate of 10 μl/min. The trap was brought in-line with the analytical column (Magic C18AQ, 3 μm 200 Å, 75 μm x 50 cm) and peptides fractionated at 300 nL/min using a segmented linear gradient: 4%–15% B (0.2% formic acid in acetonitrile) in 35 min, 15%–25% B in 65 min, 25%–50% B in 55 min. Mass spectrometry data was acquired using a data-dependent acquisition procedure with a cyclic series of a full scan acquired in Orbitrap with resolution of 60,000 followed by MS/MS (acquired in the linear ion trap) of the 20 most intense ions with a repeat count of two and a dynamic exclusion duration of 30 sec.

Peak lists in the format of MASCOT Generic Format (MGF) was generated using the Proteome Discover 1.4 (ThermoFisher). Data were searched against latest flybase *Drosophila melanogaster* protein database (madmel-all-translation-r6.03.fasta) using a local version of the Global Proteome Machine (GPM) XE Manager version 2.2.1 (Beavis Informatics Ltd., Winnipeg, Canada) with X!Tandem SLEDGEHAMMER (2013.09.01) to assign spectral data [117,118]. Precursor ion mass error tolerance was set to ±10 ppm and fragment mass error tolerance set to ±0.4 Da. Cysteine carbamidomethylation was set as a complete modification, methionine oxidation and deamidation at asparagine and glutamine residues were set as variable modifications. All LC-MS data were analyzed together in a MudPit analysis and individual data extracted to ensure that peptides that could be assigned to more than one protein were assigned consistently for all samples. The resulting identifications were filtered by peptide log GPM expectancy score (log(e)<-1.5).

### Chromatin Immunoprecipitation (ChIP) Assay

Whole-animal extracts prepared from L3 wandering larvae or white prepupae of *w* ^*1118*^ (control), *cdk8* ^*K185*^, or *cycC* ^*Y5*^ homozygous mutants were used for ChIP assays according to the protocols described previously [119]. Briefly, the fixed materials were sonicated using an Ultrasonic Processor Cell Disruptor (Branson S-450D) at 50% power output for 60 sec (2-sec-long pulse with 1 minute interval on ice). We prepared triplicate biological samples for each genotype, and for each sample, we used 2.0 μg Guinea Pig anti-USP for IP or 2.0μg normal serum isolated from the same Guinea Pig before immunization as a control. SYBR Green PCR Master Mix (Invitrogen) was used in qPCR reactions. For the qRT-PCR, the following primers were designed using Primer Express (Applied Biosystems) based on the EcR ChIP-Seq data (Kevin White’s lab): *Hsp27* F 5′-GCAACAAACAAAAGAACGGC-3′, *Hsp27* R 5′-TTTCAGAGTGCAACAGAGCTTG-3′; *E74* F 5′-TCGGTCAAAAGCAGAGTTCACA-3′, R 5′-ATTTCTCTGCAACTGCTCCC-3′; *E75* F 5′-AGGCCTGGCTGGCTGTTACTTA-3′, R 5′-CGGAGAGTTGAAGGCGAGTTT-3′; and *E78* F 5′-ATGACGTTGCCCACAAGTCATT-3′, R 5′-ACAGTTGCCTTGGCTTCTTCG-3′. Fold enrichment was calculated and normalized using the Guinea Pig normal serum as the negative control.

### Yeast Two-Hybrid Assay

To investigate physically interactions between EcR/USP and CDK8/Med14 in yeast cells, we have cloned EcR/USP into pGBKT7 (bait vector) and *Drosophila* Med14/CDK8 into pGADT7 (prey vector) (Clontech, Mountain View, CA) using the procedures described previously [120]. The following primers were used: for EcRB1-AF1 BamHIS 5′-CAGGATCCTGAAGCGGCGCTGGTCGAAC-3′ and EcRB1-AF1PstIAS 5′-CACTGCAGACCTGAAGATATAGAATTCACCGAATCGC-3′; for EcRB1-AF2 BamHIS 5′-CAGGATCCCTGATGAAATATTGGCCAAGTGTCAAGC-3′ and EcRB1-AF2 PstIAS 5′-CACTGCAGGATGGCATGAACGTCCCAGATCTC-3′; for USP-AF1 (1-100AA): dUSP-1EcoRIS 5′-CAGAATTCATGGACAACTGCGACCAGGACGC-3′ and dUSP-100BamHIAS 5′-CAGGATCCGCTGCCGCTCAGCGGATGGTT-3′; for USP-AF2 (206-509AA): dUSP-206EcoRIS 5′-CAGAATTCAGCTCTCAAGGCGGAGGAGGAGGA-3′ and dUSP-509BamHIAS 5′-CAGGATCCCTACTCCAGTTTCATCGCCAGGCC-3′; for Med14 (159-320AA): dMed14-159EcoRIS 5′-CAGAATTCCTAATTGTACATACTGTCTACATACGATCGG-3′ and dMed14-320BamHIAS 5′-CAGGATCCGGTAGTCTTTTCCTTGAGTTGAACCAC-3′.

### Statistical Analyses

At least three independent biological repeats were included for each genotype, all error bars indicate standard deviation, and *t*-tests were performed using Microsoft Excel (S1 Data). Statistical significance was shown in figures, and fold changes of 1.5 or greater were considered as biologically significant. All biochemical analyses were repeated at least three times and the representative results were shown.

## Supporting Information

S1 Data Excel spreadsheet containing the underlying numerical data and statistical analysis for Figs 1G, 1H, 2B, 2C, 3A, 3B, 3C, 3G, 5D, 5E, 6H, 7C, 7D, 7E, 7F, 7G, 7H, 7I, 7J, 7K, 8B, 8C, 8D, 8E, 8F, 8G, 8H, 9D, 9E, 9F, 9G, 9H, S2A, S2B, S6D, S6E, S7, S9A, S9B, S9C, S10A, S10B, S10C, S10D, S10E, S11A, S11B, S11C, S11D, S11E, S11F, S12B, S12C, S12D, S12E, S12F, S12G, S12H, S12I, S12J and S12K. (XLSX)

S1 Fig CycC is required for embryogenesis. Embryos from *cycC* ^*Y5*^ germline clone (*cycC* ^*Y5*^-GLC) are smaller than control (*w* ^*1118*^): (A) Dorsal and (A’) lateral views. The *cycC* ^*Y5*^-GLC embryos are embryonic lethal without proper denticle formation (C), compared to the control (B).
(TIF)

S2 Fig Further characterization and validation of the *cdk8* and *cycC* mutants. (A) Percentage of pupae with the anterior spiracle everted. The genotypes are color-coded and the number of animals for each genotype is shown. (B) The mRNA levels of *cdk8* and *cycC* were analyzed by qRT-PCR using the third instar wandering larvae. The genotypes are color-coded as in (A). * *p* < 0.05; ** *p* < 0.01 based on *t*-tests. (C) The protein levels of CDK8 and CycC were analyzed by Western blot. Underlying numerical data and statistical analysis for S2A and S2B Fig can be found in S1 Data.
(TIF)

S3 Fig Validation of the *cdk8* and *cycC* mutant alleles and the rescued lines. (A and B) Diagrams of the genomic regions of the *cdk8* (A) and *cycC* (B) loci, showing the deleted regions (hatched regions in pink); EGFP (in green) is tagged at the C-terminal ends of these two proteins. We note that the *cdk8* ^*K185*^ and *cycC* ^*Y5*^ alleles also deletes parts of their neighboring genes, i.e., *I-2*, *CG33332* and *CG3731* [50]. Although these neighboring genes are not required for viability [50], their presumed loss-of-function may contribute to molecular phenotypes that are not systematically observed both in *cdk8* ^*K185*^ and *cycC* ^*Y5*^ mutants. In general, the phenotypes are a bit stronger in *cdk8* ^*K185*^ than in *cycC* ^*Y5*^ mutants. The primers used for validation of deletions are shown in pink, and the primers used for the construction and validation of EGFP insertions are shown in green. The PCR results for insertions (C) and deletions (D) using the genomic DNA from the following genotypes: *a*) *w* ^*1118*^; *b*) *cdk8* ^*+*^ *-EGFP; cdk8* ^*K185*^; *c*) *cycC* ^*+*^ *-EGFP; cycC* ^*Y5*^; *d*) *cdk8* ^*K185*^; *e*) *cdk8* ^*K185*^, *cycC* ^*Y5*^; and *f*) *cycC* ^*Y5*^. The molecular weight marker used was the 1 kb Plus DNA Ladder (Invitrogen). Note that the upper band (approximately 2,500 bp) in “*cdk8* ^*+*^ *-EGFP; cdk8* ^*k185”*^ is amplified from the *cdk8* ^*+*^ *-EGFP* insertion as the template (‘***’ in D). For validation of the deletion in *cdk8* ^*K185*^ mutants, the following primers were used: *cdk8-K185F*: *5′-*TGTGGGCTGGGATTGTTCTGC, and *cdk8-K185R*: 5′-ACATCTGGGCTATTGGCTGTATTTTCG. The expected product sizes are 1792bp for control, 910bp for *cdk8* ^*K185*^ deletion, and 2500bp for *cdk8* ^*+*^ *-EGFP* insertion. For the verification of deletion in *cycC* ^*Y5*^ line, the following primers were used: *cycC-Y5F*: 5′-TGGTCCTCTGCCAAATGCCAGTC, and *cycC-Y5R*: 5′-TGGAGGAGCGGATTCTGTTGTAGTCG. The expected product sizes are 3989bp for control and 1256bp for *cycC* ^*Y5*^ deletion. For the verification of insertion of EGFP in rescued *cdk8* line (*cdk8* ^*+*^ *-EGFP; cdk8* ^*K185*^): *cdk8 5*.*11*: 5′-GCAGCAAATGAACGCTGAG and *cdk8 IN-4(EGFP)*: 5′-TGTATCAGTCTCTCACTTGTACAGCTCGTCCATGCCG, and the expected product size is 931bp. For verification of the insertion of EGFP in rescued *cycC* line (*cycC* ^*+*^ *-EGFP/CyO; cycC* ^*Y5*^): *cycC 5*.*11*: 5′-ATCGCTATAGCCTGCCTTCA and *cycC IN-4(EGFP)*: 5′- TATGAAGCTCTTCTACTTGTACAGCTCGTCCATGCCG, and the expected product size is 960bp.
(TIF)

S4 Fig Supplying the EcR ligand 20E in food did not rescue the aberrant morphology of the *cdk8* and *cycC* mutants. (A) 2 μM of 20E; (B) 20 μM of 20E; (C) 100 μM of 20E; (D) 2 mM of 20E. With 20 E treatment, all of the *cdk8* and *cycC* mutants still had the same defective pupal morphology, delayed pupariation, and prepupal lethality.
(TIF)

S5 Fig Specificity of antisera against USP and SREBP. (A) Ectopic expression of USP can be detected by the ployclonal antibody generated in guinea pig. Together with the control (*w* ^*1118*^), the mid-L3 *P[hs-neo* ^*R*^, *hs-usp]/TM3* larvae were heat-shocked at 37 ˚C for 2 hr, recovered at 25 ˚C for 12 hr and then collected for Western blot. (B) Ectopic expression of mature nuclear form of SREBP (approximately 49 kDa) recognized by the anti-SREBP antibody. The predicted size of mature nuclear form of SREBP (the first 451AA of SREBP; [121]) is 49.4 kDa. (C) The SREBP in L3 wandering larvae was depleted by *ubi-Gal4*-driven expression of short hairpin RNA target *SREBP* (*TRiP*.*HMS00080* line). The predicted size of full-length SREBP (1113 AA) is 124.5 kDa, and the upper band (53–54 kDa) in the middle panel is likely the N-terminal fragment of SREBP after cleavage by the S1P (site 1 protease), both of which were reduced by RNAi.
(TIF)

S6 Fig The distribution of EcR and USP in *cdk8* or *cycC* mutants. (A-C) Immunostaining of EcR (A, B, and C) and USP (A’, B’, and C’) show that they are enriched in the nuclei of the wild-type (*w* ^*1118*^), *cdk8* ^*K185*^, and *cycC* ^*Y5*^ mutant salivary gland cells. The nuclei are stained with the DNA-binding dye DAPI (A”, B”, and C”). Scale bar in (C”): 20μm. (D) Quantification of the EcR levels in nucleus and cytoplasm from immunostaining using ImageJ. (E) Quantification of the USP levels in nucleus and cytoplasm from immunostaining using ImageJ. The genotypes are color coded as shown in (D). * *p* < 0.05; ** *p* < 0.01 based on *t*-tests. Underlying numerical data and statistical analysis for S6D and S6E Fig can be found in S1 Data.
(TIF)

S7 Fig The relative mRNA levels of *dE2f1*, *Dp*, and several E2F1 target genes in *cdk8* and *cycC* mutants at L3 wandering stage analyzed by qRT-PCR assay. * *p* < 0.05; ** *p* < 0.01 based on *t*-tests. Underlying numerical data and statistical analysis for S7 Fig can be found in S1 Data.
(TIF)

S8 Fig (A) The two LXXLL motifs in Med1 are conserved in vertebrates, but not in flies and worms. (B) A model for Mediator complexes in regulating EcR-dependent gene expression. We propose that both CDK8 submodule (via CDK8) and the small Mediator complex (via Med14) interact with EcR, and the mediator complexes are required for EcR-USP to indirectly interact with the general transcription factors (GTFs) and RNA Pol II. In this model, the Mediators complexes serve as a molecular bridge between EcR-USP and the general transcription machinery. See the Discussion for more details.
(TIF)

S9 Fig The relative mRNA levels of *cdk8* and *cycC* (A), *E74* (B), and *dACC* (C) at different developmental stages analyzed by qRT-PCR. The different stages in (A) are the same as in Fig 4A. * *p* < 0.05; ** *p* < 0.01 based on *t*-tests. Underlying numerical data and statistical analysis for S9A, S9B, and S9C Fig can be found in S1 Data.
(TIF)

S10 Fig The effects of starvation on gene expression, ecdysteroid titer, and pupal size. The effects of starvation on the expression of *cycC* (A), *usp* (B), *E75* (C), and *E78* (D), as assayed by qRT-PCR. (E) The effect of starvation on the biosynthesis of ecdysteroid measured by ELISA. The *x*-axis represents the number of hours AEL. (F) The effects of starvation and refeeding of wild-type larvae after they have reached critical weight on pupal sizes. The schemes for treatment were described in Fig 8H. Underlying numerical data and statistical analysis for S10A, S10B, S10C, S10D and S10E Fig can be found in S1 Data.
(TIF)

S11 Fig The effects of refeeding of starved larvae on the expression of *cycC* (A), *usp* (B), *E74* (C), *E75* (D), *E78* (E) and *dACC* (F), as assayed by qRT-PCR. The *x*-axis represents the number of hours for refeeding. * *p* < 0.05; ** *p* < 0.01 based on *t*-tests. Underlying numerical data and statistical analysis for S11A, S11B, S11C, S11D, S11E and S11F Fig can be found in S1 Data.
(TIF)

S12 Fig The effects of refeeding of starved larvae on the CDK8-SREBP/EcR regulatory network. (A) The protein levels of CycC and SREBP in starved versus refed larvae after 3, 6, 9 hr of refeeding. The control (anti-actin) is the same as the Fig 9C. (B–K) The mRNA levels of *cdk8*, *cycC*, *EcR*, *usp*, *E75*, *E78*, *SREBP*, *dFAS*, *dACC*, and *dACS* in starved versus refed larvae after 3, 6, 9 hr of refeeding. The *x*-axis represents the number of hours for refeeding. * *p* < 0.05; ** *p* < 0.01 based on *t*-tests. Underlying numerical data and statistical analysis for S12B, S12C, S12D, S12E, S12F, S12G, S12H, S12I, S12J and S12K Fig can be found in S1 Data.
(TIF)

S1 Table Effects of *cdk8* or *cycC* mutation on the expression levels of factors involved in the ecdysone signaling. The fold changes (*cdk8/control* and *cycC/control*) were calculated based on the microarray analyses of the *cdk8* and *cycC* mutant L3 wandering larvae compared to the control (*w* ^*1118*^) larvae. The similar results were visually showed in Fig 2A.
(XLSX)

S2 Table Known EcR-USP cofactors identified by the LC-MS/MS analysis. (XLSX)

S3 Table Primer sequences for the qRT-PCR assays. (XLSX)

## Abbreviations:

20E: 20-hydroxyecdysone
ACC: acetyl-CoA carboxylase
ACS: acetyl-CoA synthase
AEL: after egg laying
AF1: activation function domain 1
APF: after puparium formation
BAC: bacterial artificial chromosome
br: broad
CDK8: cyclin-dependent kinase 8
ChIP: chromatin immunoprecipitation
CycC: Cyclin C
CycE: Cyclin E
DAPI: 4′,6-Diamidino-2-phenylindole dihydrochloride
DBD: DNA-binding domain
dib: disembodied
dDOR: *Drosophila* Diabetes and Obesity Regulated
dILPs: *Drosophila* insulin-like peptides
DTT: dithiothreitol
EcR: Ecdysone Receptor
EcRE: ecdysone response element
FAS: Fatty acid synthase
βFtz-F1: Ftz transcription factor 1
Hsp27: Heat shock protein 27
ImpE2: Ecdysone-inducible gene E2
JH: juvenile hormone
Jheh1: JH-epoxide hydrolase
Jhl-26: JH-inducible protein 26
kni: knirps
kto: kohtalo or Med12
LBD: ligand-binding domain
LC-MS/MS: liquid chromatography-tandem mass spectrometry
mld: molting defective
nvd: neverland
PBS: phosphate buffered saline
PG: prothoracic gland
phm: phantom
PMSF: phenylmethanesulfonylfluoride
Pol II: RNA polymerase II
PTTH: prothoracicotropic hormone
qRT-PCR: quantitative reverse-transcription polymerase chain reaction
Rig: Rigor mortis
Rp49: ribosomal protein 49
RXR: Retinoid X Receptor
sad: shadow
Sgs1: Salivary gland secretion 1
shd: shade
skd: skuld or Med13
Smr: Smrter
spo: spook
spok: spookier
SREBP: sterol regulatory element binding protein
STAT: signal-transducer and activator of transcription protein
TGF-β: transforming growth factor β
TOR: target of rapamycin
TRR: Trithorax-related
USP: Ultraspiracle
vvl: ventral veins lacking
WPP: white prepupa
