# Correction: CDK8-Cyclin C Mediates Nutritional Regulation of Developmental Transitions through the Ecdysone Receptor in *Drosophila*

**DOI:** 10.1371/journal.pbio.1002250

**Published:** 2015-08-25

**Authors:** The PLOS Biology Staff

There is an error in affiliation 2 for authors Wu Xu and Liying Huang. Affiliation 2 should be: Department of Chemistry, University of Louisiana at Lafayette, Lafayette, Louisiana, United States of America.
