# Supplementary figures and images for: CDK8-Cyclin C Mediates Nutritional Regulation of Developmental Transitions through the Ecdysone Receptor in Drosophila
Source: PLoS Biol. 2015 Jul 29;13(7):e1002207. doi: 10.1371/journal.pbio.1002207 (PMC4519132; doi:10.1371/journal.pbio.1002207)

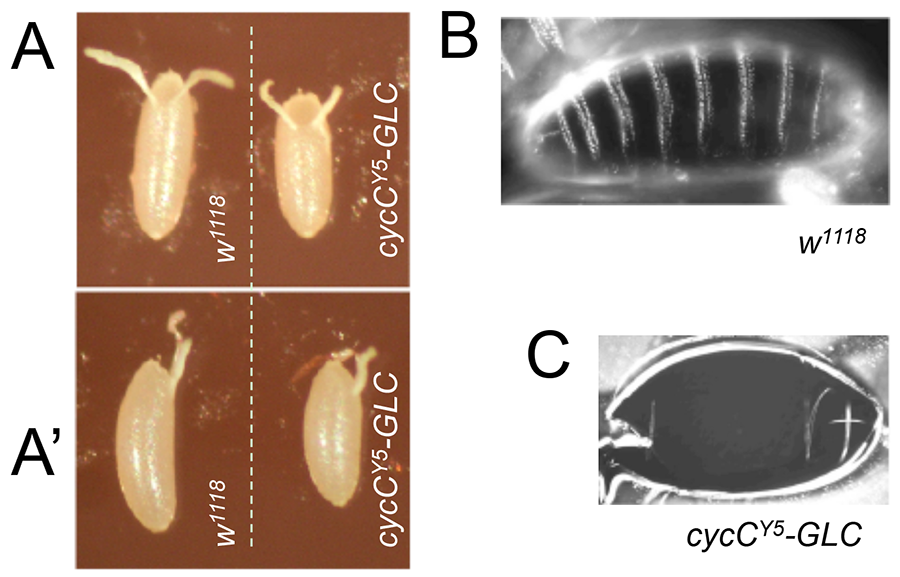

Supplement: S1 Fig — Embryos from cycC Y5 germline clone (cycC Y5-GLC) are smaller than control (w 1118): (A) Dorsal and (A’) lateral views. The cycC Y5-GLC embryos are embryonic lethal without proper denticle formation (C), compared to the control (B). (TIF) [file pbio.1002207.s002.tif]

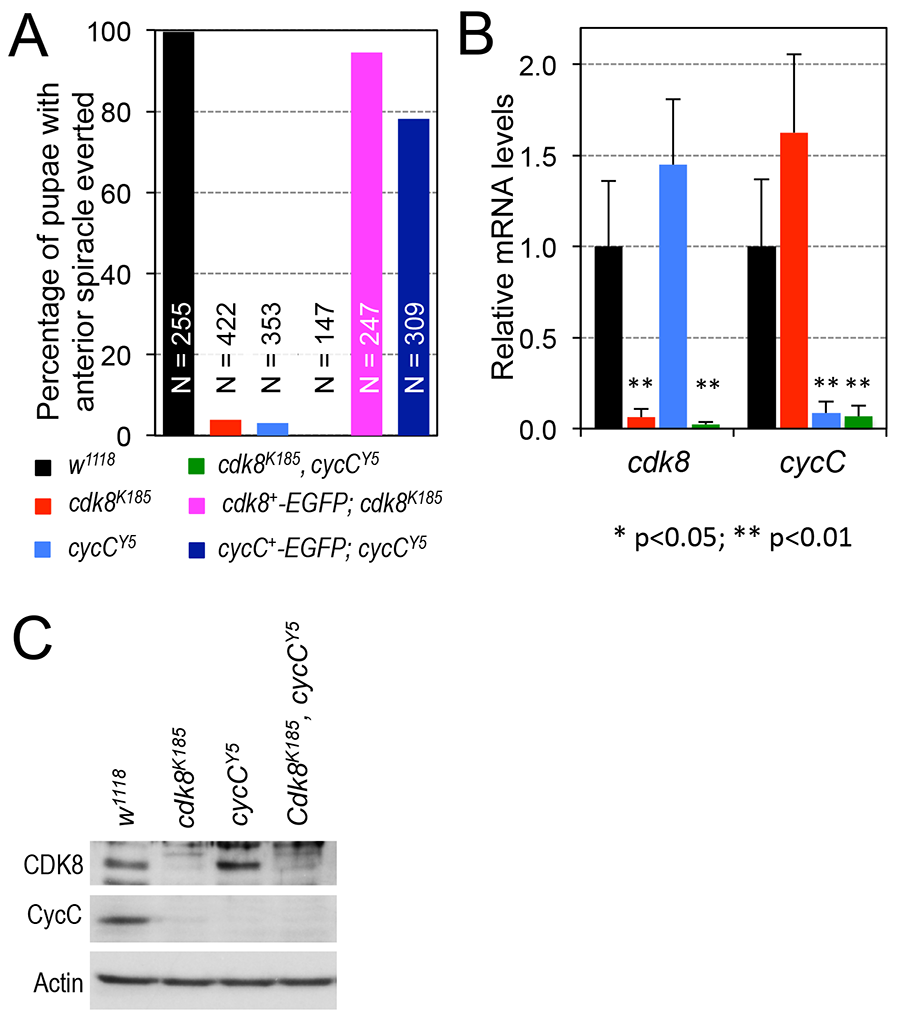

Supplement: S2 Fig — (A) Percentage of pupae with the anterior spiracle everted. The genotypes are color-coded and the number of animals for each genotype is shown. (B) The mRNA levels of cdk8 and cycC were analyzed by qRT-PCR using the third instar wandering larvae. The genotypes are color-coded as in (A). * p < 0.05; ** p < 0.01 based on t-tests. (C) The protein levels of CDK8 and CycC were analyzed by Western blot. Underlying numerical data and statistical analysis for S2A and S2B Fig can be found in S1 Data. (TIF) [file pbio.1002207.s003.tif]

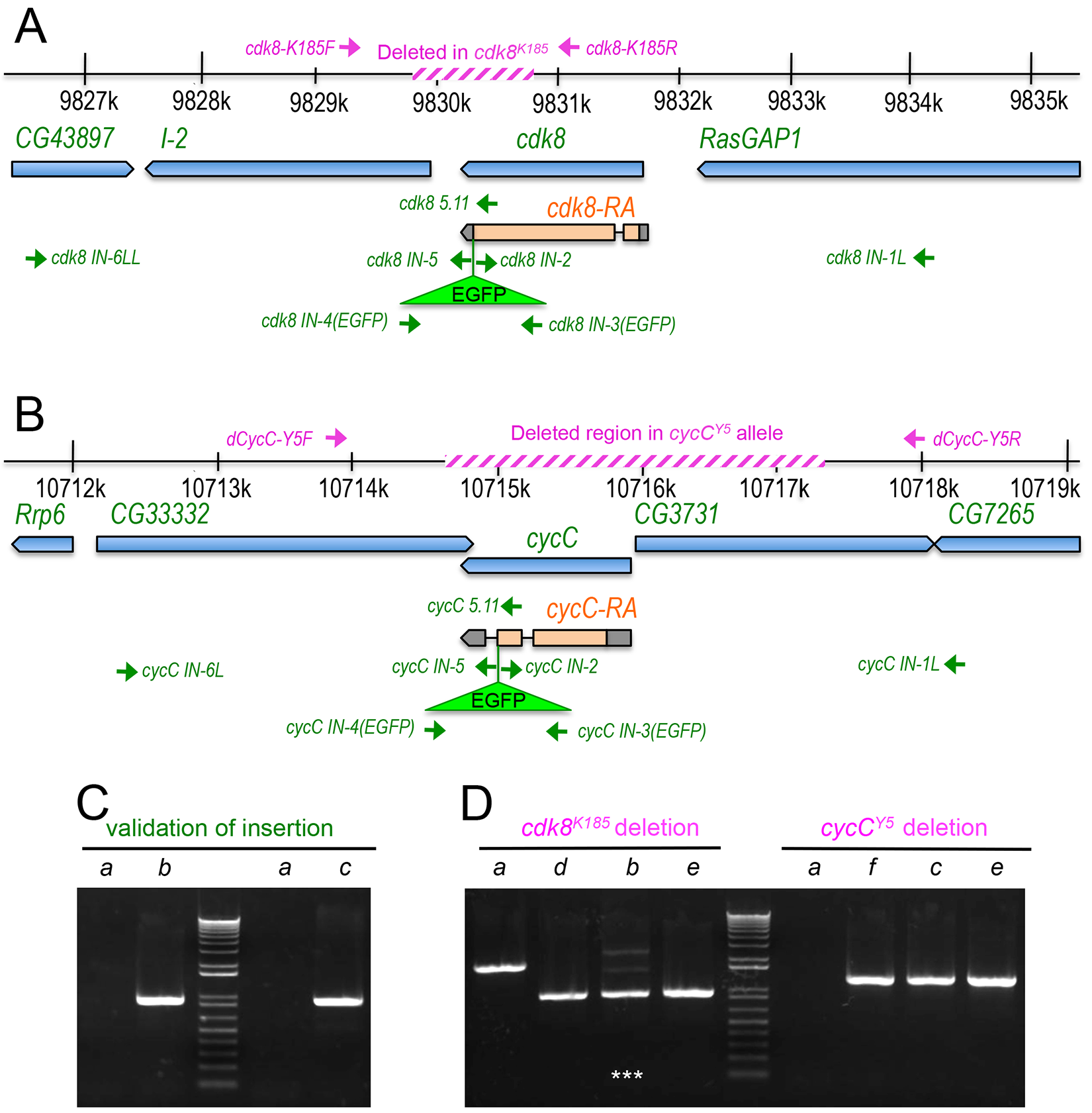

Supplement: S3 Fig — (A and B) Diagrams of the genomic regions of the cdk8 (A) and cycC (B) loci, showing the deleted regions (hatched regions in pink); EGFP (in green) is tagged at the C-terminal ends of these two proteins. We note that the cdk8 K185 and cycC Y5 alleles also deletes parts of their neighboring genes, i.e., I-2, CG33332 and CG3731 [50]. Although these neighboring genes are not required for viability [50], their presumed loss-of-function may contribute to molecular phenotypes that are not systematically observed both in cdk8 K185 and cycC Y5 mutants. In general, the phenotypes are a bit stronger in cdk8 K185 than in cycC Y5 mutants. The primers used for validation of deletions are shown in pink, and the primers used for the construction and validation of EGFP insertions are shown in green. The PCR results for insertions (C) and deletions (D) using the genomic DNA from the following genotypes: a) w 1118; b) cdk8 + -EGFP; cdk8 K185; c) cycC + -EGFP; cycC Y5; d) cdk8 K185; e) cdk8 K185, cycC Y5; and f) cycC Y5. The molecular weight marker used was the 1 kb Plus DNA Ladder (Invitrogen). Note that the upper band (approximately 2,500 bp) in “cdk8 + -EGFP; cdk8 k185” is amplified from the cdk8 + -EGFP insertion as the template (‘***’ in D). For validation of the deletion in cdk8 K185 mutants, the following primers were used: cdk8-K185F: 5′-TGTGGGCTGGGATTGTTCTGC, and cdk8-K185R: 5′-ACATCTGGGCTATTGGCTGTATTTTCG. The expected product sizes are 1792bp for control, 910bp for cdk8 K185 deletion, and 2500bp for cdk8 + -EGFP insertion. For the verification of deletion in cycC Y5 line, the following primers were used: cycC-Y5F: 5′-TGGTCCTCTGCCAAATGCCAGTC, and cycC-Y5R: 5′-TGGAGGAGCGGATTCTGTTGTAGTCG. The expected product sizes are 3989bp for control and 1256bp for cycC Y5 deletion. For the verification of insertion of EGFP in rescued cdk8 line (cdk8 + -EGFP; cdk8 K185): cdk8 5.11: 5′-GCAGCAAATGAACGCTGAG and cdk8 IN-4(EGFP): 5′-TGTATCAGTCTCTCACTTGTACAGCTCGTCCATGCCG, and the expected product [file pbio.1002207.s004.tif]

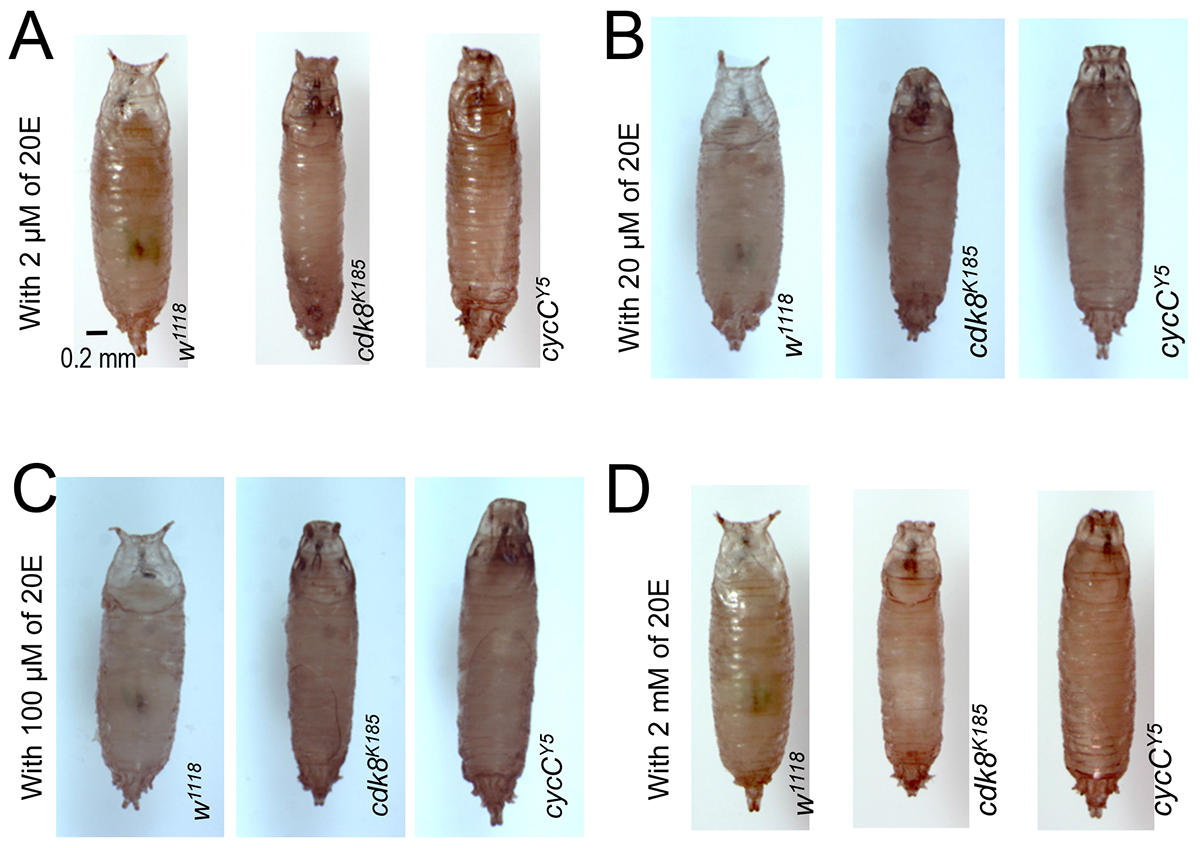

Supplement: S4 Fig — (A) 2 μM of 20E; (B) 20 μM of 20E; (C) 100 μM of 20E; (D) 2 mM of 20E. With 20 E treatment, all of the cdk8 and cycC mutants still had the same defective pupal morphology, delayed pupariation, and prepupal lethality. (TIF) [file pbio.1002207.s005.tif]

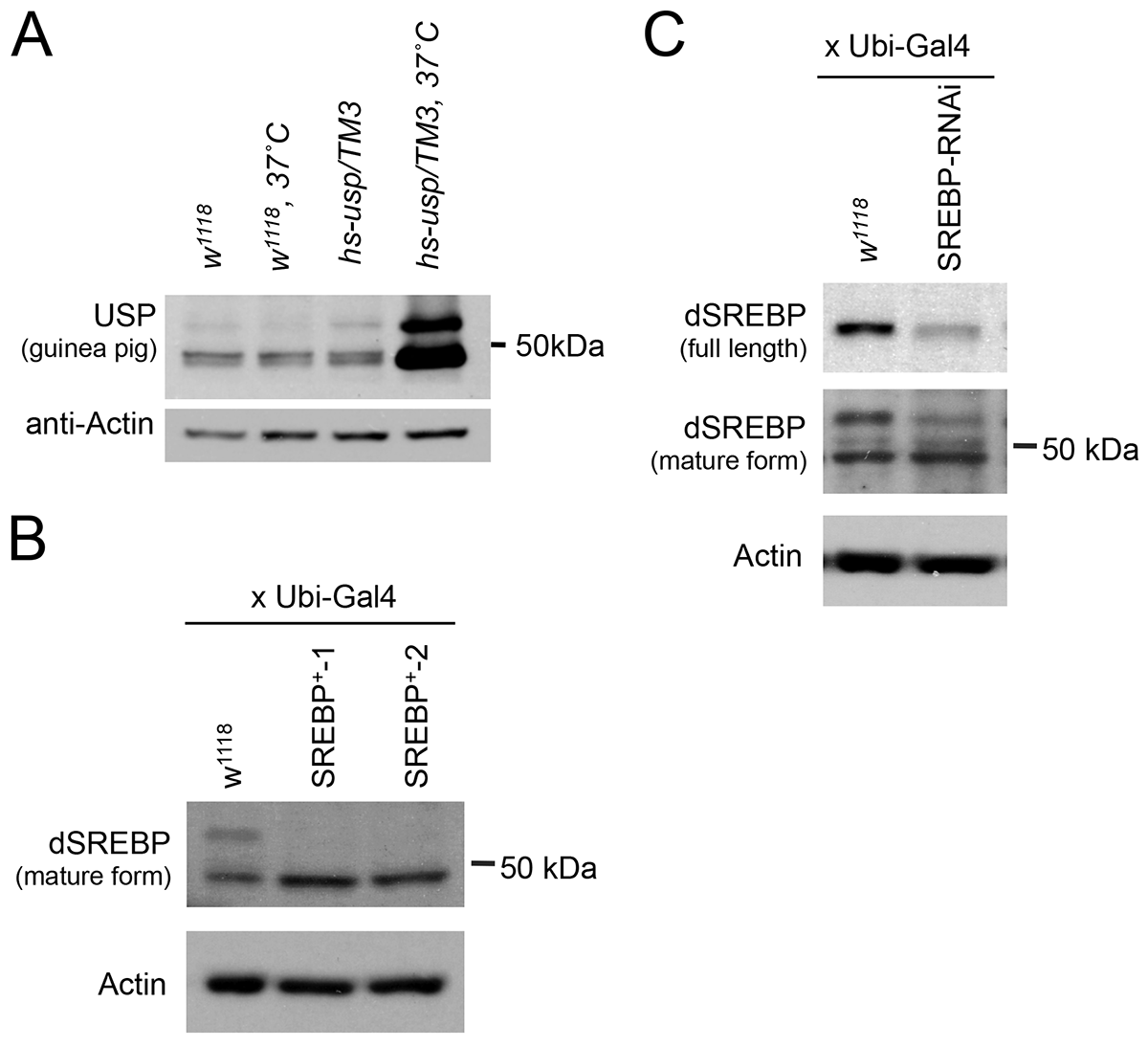

Supplement: S5 Fig — (A) Ectopic expression of USP can be detected by the ployclonal antibody generated in guinea pig. Together with the control (w 1118), the mid-L3 P[hs-neo R, hs-usp]/TM3 larvae were heat-shocked at 37 ˚C for 2 hr, recovered at 25 ˚C for 12 hr and then collected for Western blot. (B) Ectopic expression of mature nuclear form of SREBP (approximately 49 kDa) recognized by the anti-SREBP antibody. The predicted size of mature nuclear form of SREBP (the first 451AA of SREBP; [121]) is 49.4 kDa. (C) The SREBP in L3 wandering larvae was depleted by ubi-Gal4-driven expression of short hairpin RNA target SREBP (TRiP.HMS00080 line). The predicted size of full-length SREBP (1113 AA) is 124.5 kDa, and the upper band (53–54 kDa) in the middle panel is likely the N-terminal fragment of SREBP after cleavage by the S1P (site 1 protease), both of which were reduced by RNAi. (TIF) [file pbio.1002207.s006.tif]

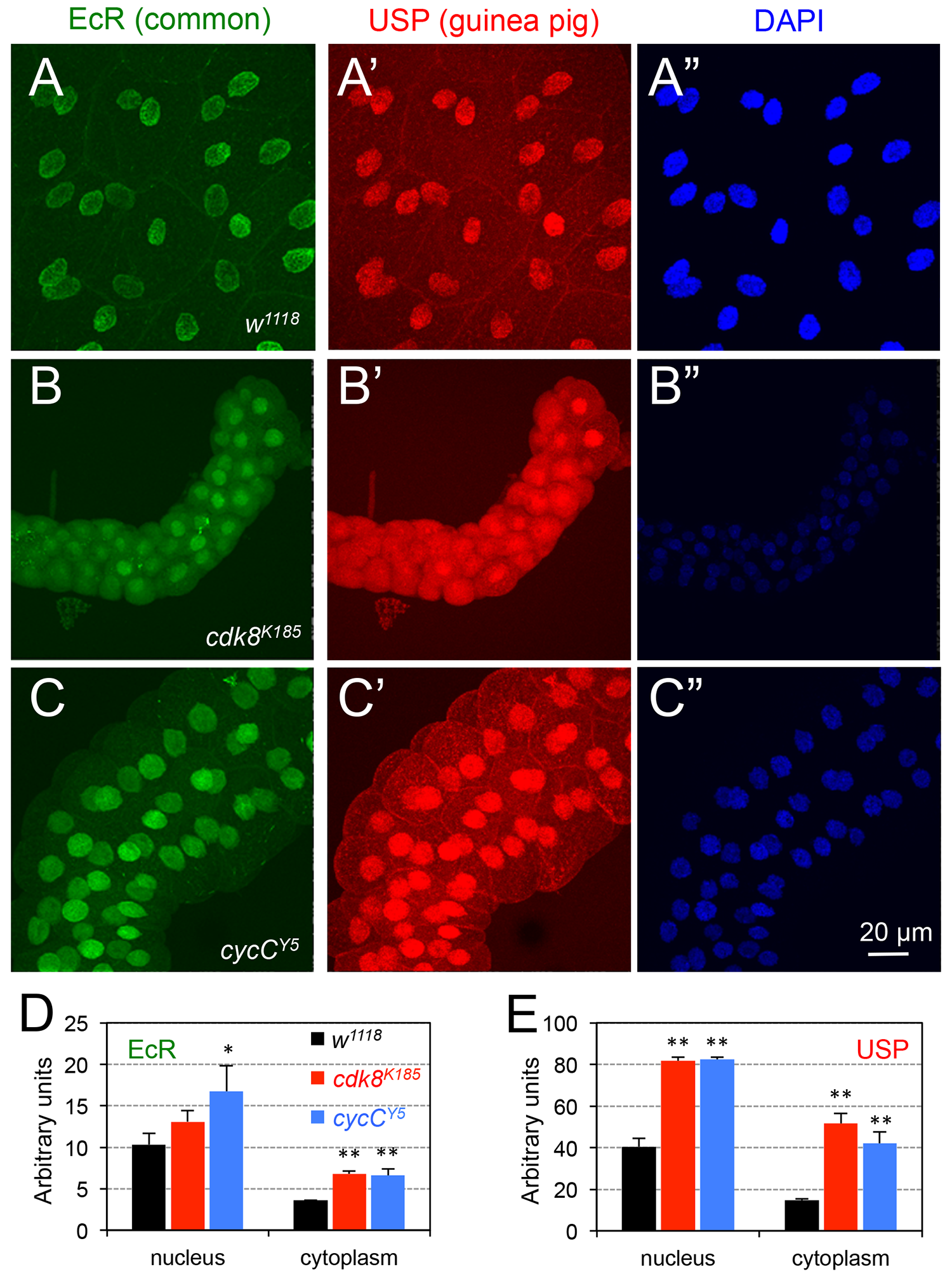

Supplement: S6 Fig — (A-C) Immunostaining of EcR (A, B, and C) and USP (A’, B’, and C’) show that they are enriched in the nuclei of the wild-type (w 1118), cdk8 K185, and cycC Y5 mutant salivary gland cells. The nuclei are stained with the DNA-binding dye DAPI (A”, B”, and C”). Scale bar in (C”): 20μm. (D) Quantification of the EcR levels in nucleus and cytoplasm from immunostaining using ImageJ. (E) Quantification of the USP levels in nucleus and cytoplasm from immunostaining using ImageJ. The genotypes are color coded as shown in (D). * p < 0.05; ** p < 0.01 based on t-tests. Underlying numerical data and statistical analysis for S6D and S6E Fig can be found in S1 Data. (TIF) [file pbio.1002207.s007.tif]

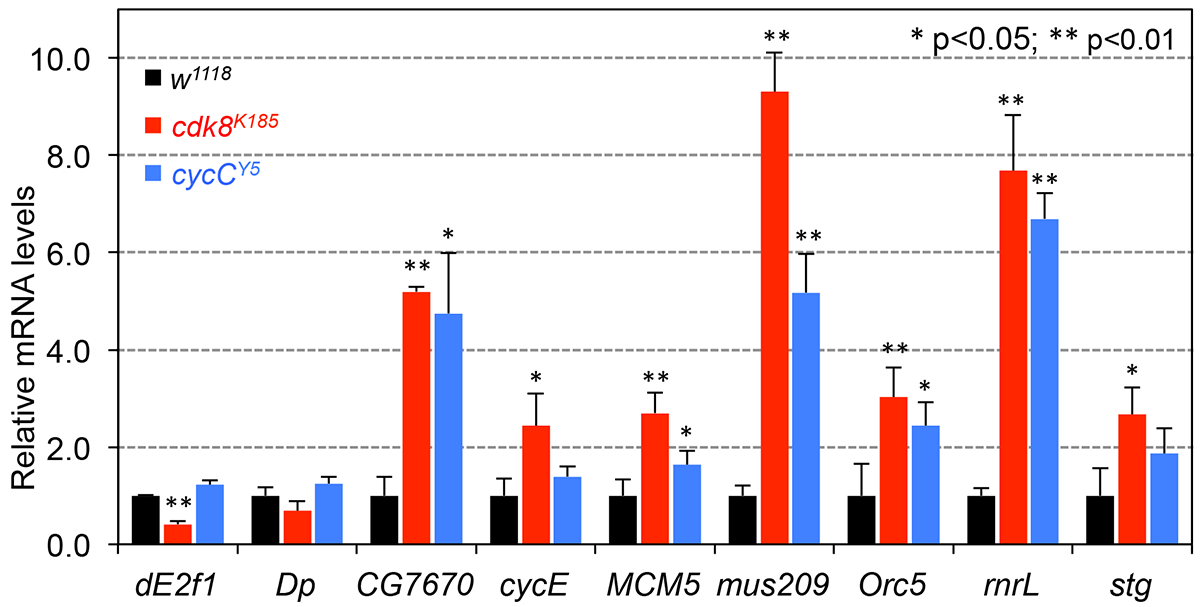

Supplement: S7 Fig — * p < 0.05; ** p < 0.01 based on t-tests. Underlying numerical data and statistical analysis for S7 Fig can be found in S1 Data. (TIF) [file pbio.1002207.s008.tif]

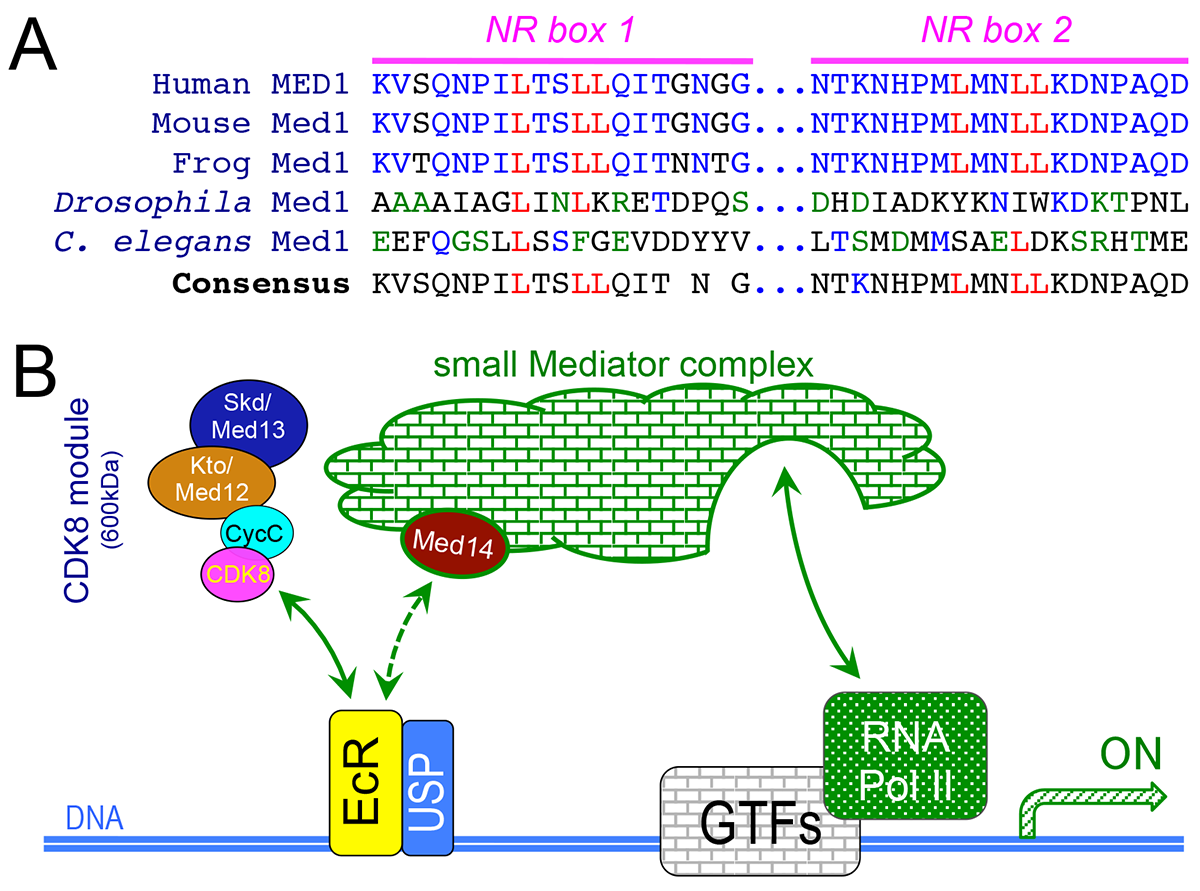

Supplement: S8 Fig — (B) A model for Mediator complexes in regulating EcR-dependent gene expression. We propose that both CDK8 submodule (via CDK8) and the small Mediator complex (via Med14) interact with EcR, and the mediator complexes are required for EcR-USP to indirectly interact with the general transcription factors (GTFs) and RNA Pol II. In this model, the Mediators complexes serve as a molecular bridge between EcR-USP and the general transcription machinery. See the Discussion for more details. (TIF) [file pbio.1002207.s009.tif]

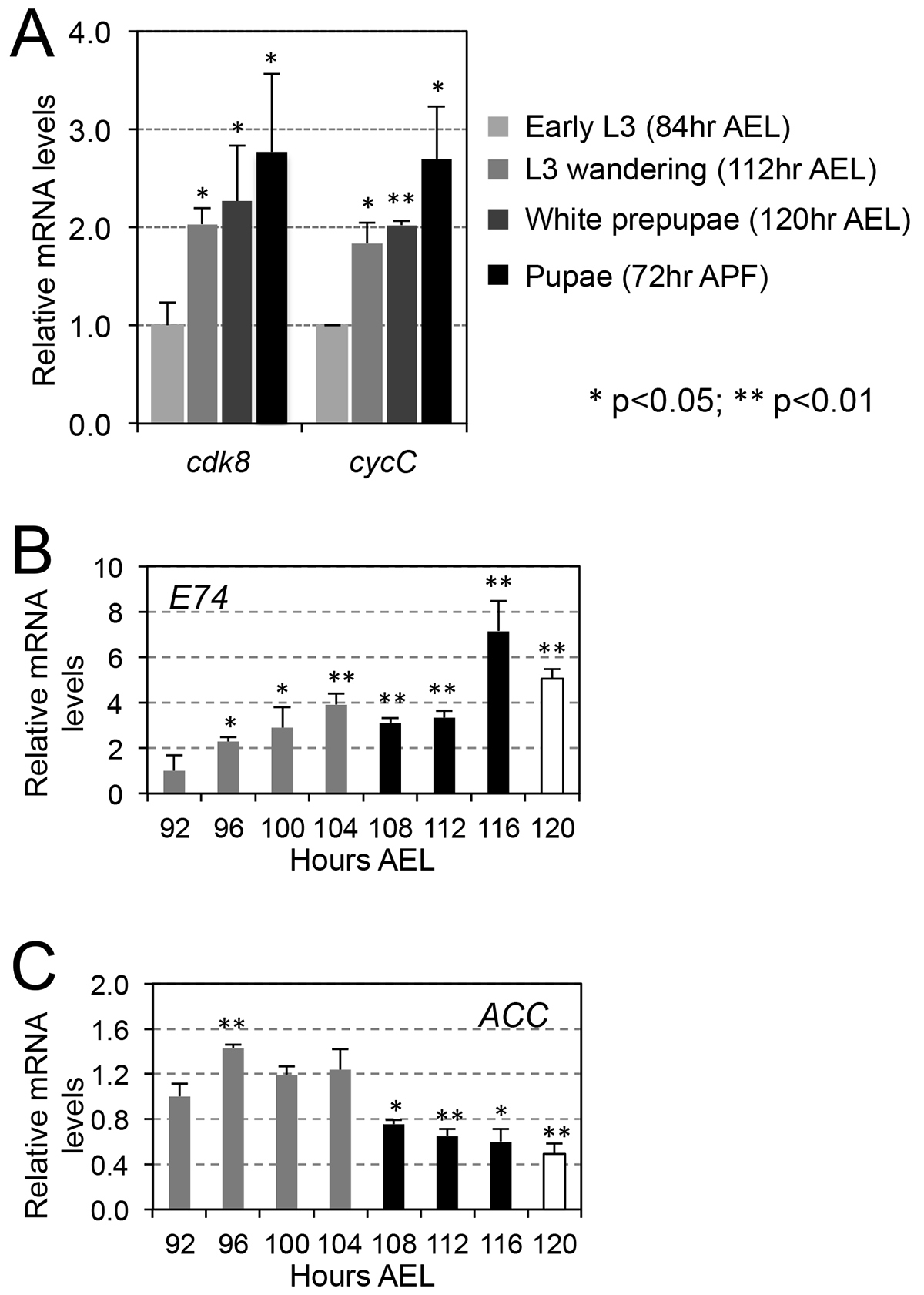

Supplement: S9 Fig — The different stages in (A) are the same as in Fig 4A. * p < 0.05; ** p < 0.01 based on t-tests. Underlying numerical data and statistical analysis for S9A, S9B, and S9C Fig can be found in S1 Data. (TIF) [file pbio.1002207.s010.tif]

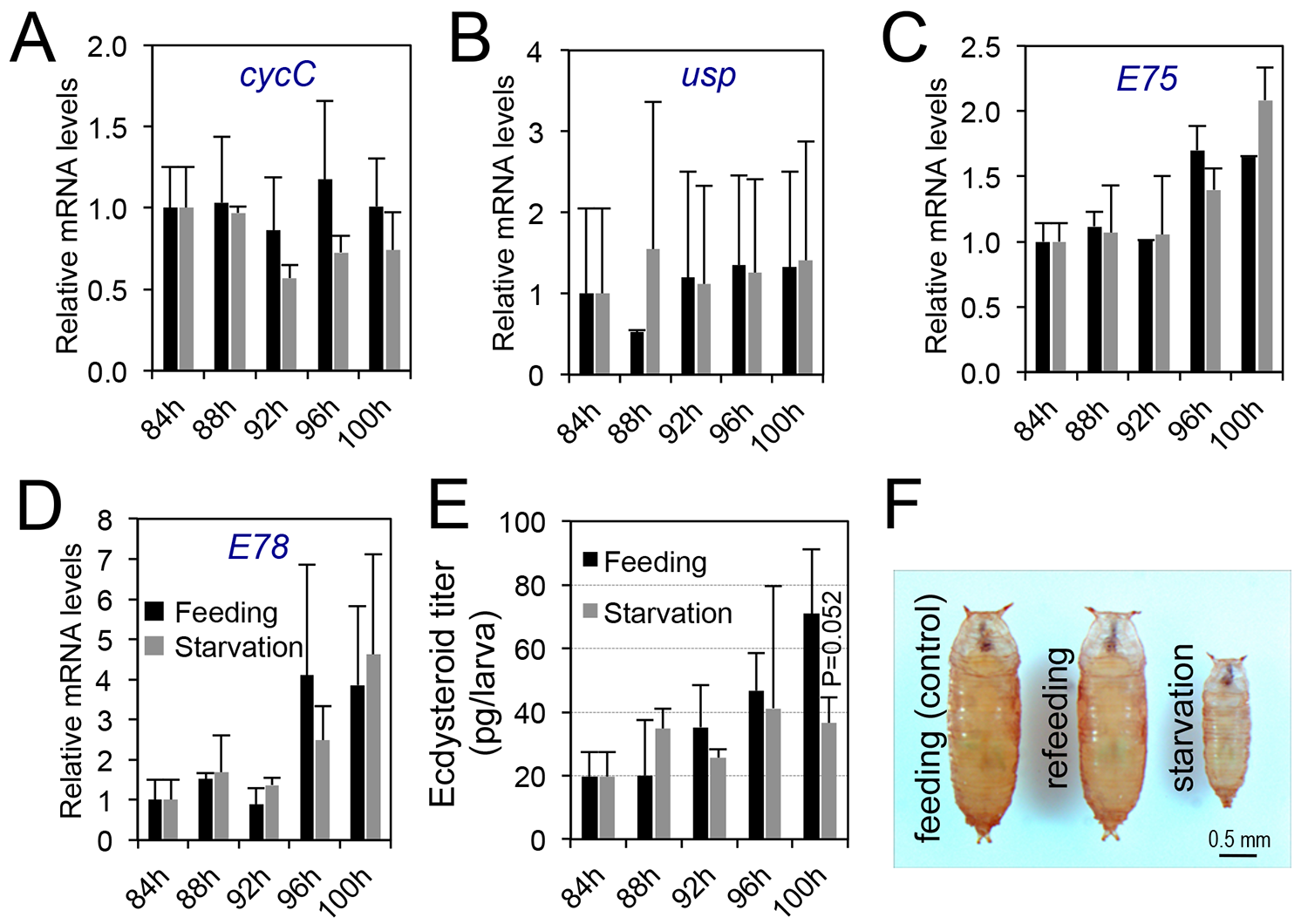

Supplement: S10 Fig — The effects of starvation on the expression of cycC (A), usp (B), E75 (C), and E78 (D), as assayed by qRT-PCR. (E) The effect of starvation on the biosynthesis of ecdysteroid measured by ELISA. The x-axis represents the number of hours AEL. (F) The effects of starvation and refeeding of wild-type larvae after they have reached critical weight on pupal sizes. The schemes for treatment were described in Fig 8H. Underlying numerical data and statistical analysis for S10A, S10B, S10C, S10D and S10E Fig can be found in S1 Data. (TIF) [file pbio.1002207.s011.tif]

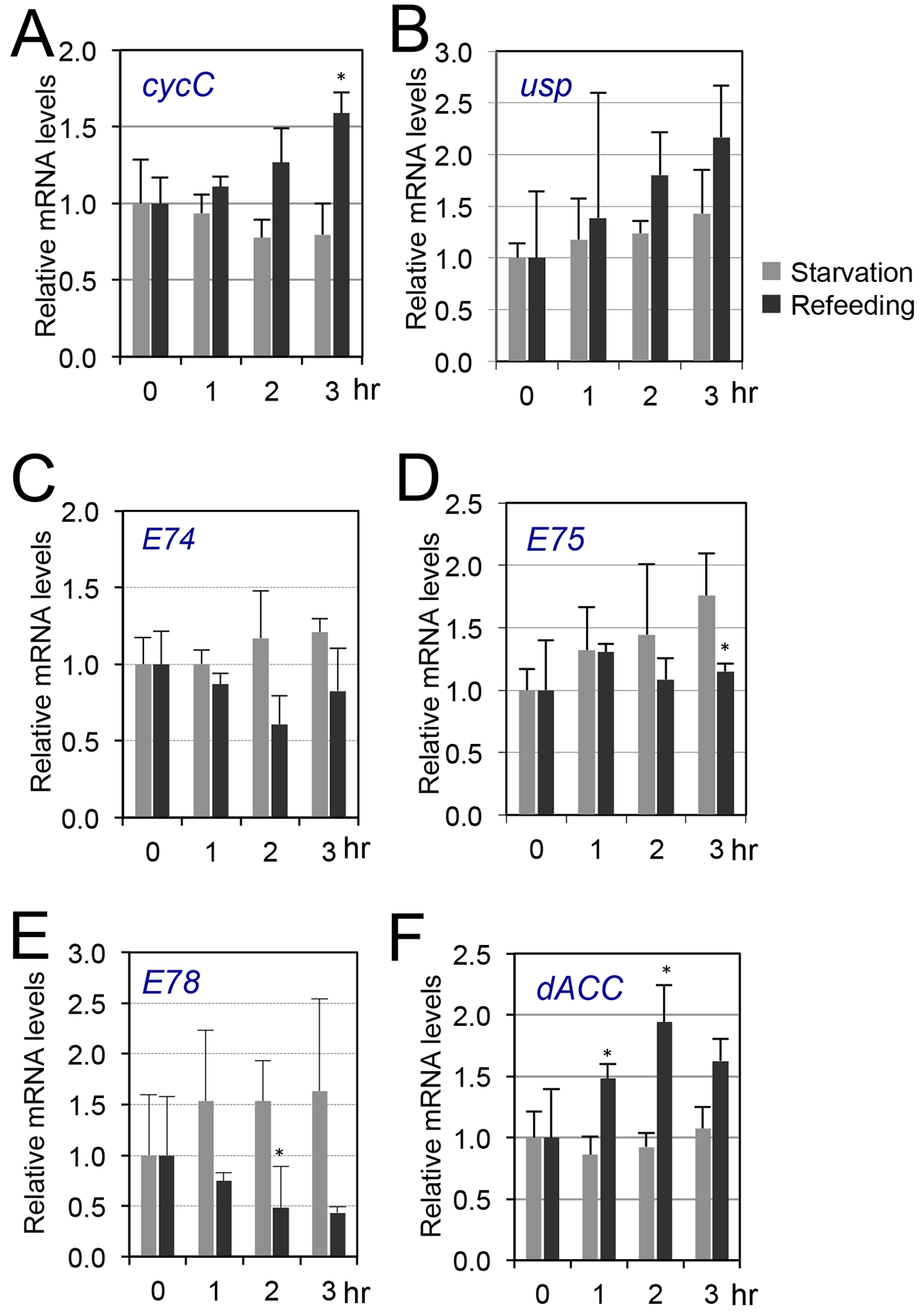

Supplement: S11 Fig — The x-axis represents the number of hours for refeeding. * p < 0.05; ** p < 0.01 based on t-tests. Underlying numerical data and statistical analysis for S11A, S11B, S11C, S11D, S11E and S11F Fig can be found in S1 Data. (TIF) [file pbio.1002207.s012.tif]

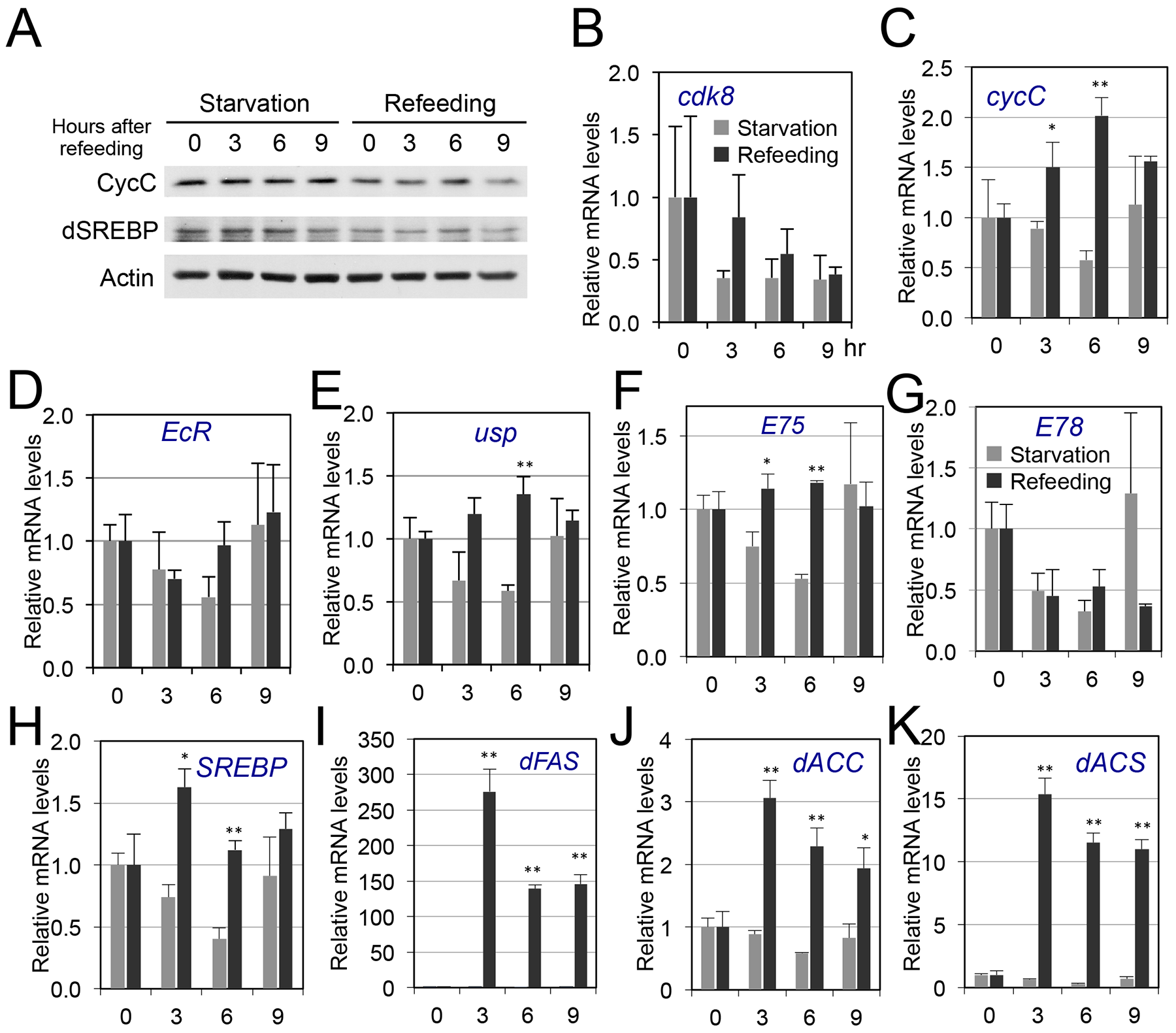

Supplement: S12 Fig — (A) The protein levels of CycC and SREBP in starved versus refed larvae after 3, 6, 9 hr of refeeding. The control (anti-actin) is the same as the Fig 9C. (B–K) The mRNA levels of cdk8, cycC, EcR, usp, E75, E78, SREBP, dFAS, dACC, and dACS in starved versus refed larvae after 3, 6, 9 hr of refeeding. The x-axis represents the number of hours for refeeding. * p < 0.05; ** p < 0.01 based on t-tests. Underlying numerical data and statistical analysis for S12B, S12C, S12D, S12E, S12F, S12G, S12H, S12I, S12J and S12K Fig can be found in S1 Data. (TIF) [file pbio.1002207.s013.tif]
